# An Interactive Multimodality Curriculum Teaching Medicine Residents About Oncologic Documentation and Billing

**DOI:** 10.15766/mep_2374-8265.10746

**Published:** 2018-08-30

**Authors:** Arpan Patel, Azka Ali, Forat Lutfi, Adeaze Nwosu-lheme, Merry Jennifer Markham

**Affiliations:** 1 Hematology/Oncology Fellow, Division of Hematology & Oncology, University of Florida College of Medicine; 2 Medical Resident, Department of Medicine, University of Florida College of Medicine; 3 Hematology/Oncology Fellow, Division of Hematology & Oncology, Department of Medicine, University of Florida College of Medicine; 4 Associate Director, Medical Affairs, University of Florida Health Cancer Center; 5 Associate Professor, Division of Hematology & Oncology, Department of Medicine, University of Florida College of Medicine

**Keywords:** Oncology, Coding, Billing, Documentation, Simulation, Internal Medicine, Didactics

## Abstract

**Introduction:**

Physicians recognize the importance of clinical documentation for accuracy of coding and billing, but it is emphasized little in residency curricula, with an even smaller emphasis on oncology-specific documentation. We developed an educational curriculum to teach residents about clinical documentation for cancer patients. Our tool kit includes didactics, simulated history and physical (H&P) documentation, and personal feedback.

**Methods:**

A preintervention survey was first administered to gauge baseline knowledge. A simulated H&P was developed that required participants to complete their own assessment and plan. We delivered a 25-minute lecture regarding billing and coding along with documentation tips and tricks specific to hematology/oncology. Thereafter, we handed out a second H&P, and participants had to once again complete their own assessment and plan. These H&Ps were graded by three reviewers using a rubric. We then gave residents personalized feedback using the above data and administered a postintervention survey.

**Results:**

The postintervention survey revealed that 100% of the residents surveyed found this activity helpful, 83% noted that further knowledge of diagnosis codes was helpful to their learning, 100% noted that that this activity taught them to improve documentation, 91% said they were more likely to use cancer-specific diagnoses, and 91% said they would benefit from direct feedback-based education.

**Discussion:**

Didactic and formal education is more effective when combined with hands-on examples and direct personalized feedback.

## Educational Objectives

By the end of this activity, learners will be able to:

1. Accurately document and code oncologic patient encounters.
2. Actively apply comprehensive medical documentation skills to a mock oncology clinical encounter.
3. Distinguish important aspects of medical documentation to ensure appropriate documentation.
4. Identify crucial components of a history and physical exam to ensure an appropriate level of diagnosis-related group code.

## Introduction

En route to becoming an independent practicing physician, one must go through rigorous training after medical school, including residency and an optional fellowship. Along with patient care information, physicians are also responsible for documentation and accurate coding of clinical encounters.^1^ The Accreditation Council for Graduate Medical Education has stated that a larger context is part of the responsibility of the physician, and billing and coding are necessary knowledge in postresidency and fellowship training. Due to the challenges of patient volume, medical complexity, and an immense focus on optimizing hospital efficiency, learners often overlook accurate clinical documentation that captures the complexity of the medical encounter. While Medicare does not distinguish between overcoding and underdocumentation, Medicaid has strict regulations for reimbursement if a service is not supported by documentation.^2^ It is important for resident physicians to receive training about appropriate documentation, billing, and coding because they ultimately affect reimbursements and health care costs to patients, as well as improving communication between health care professionals.

Currently, billing and coding are taught primarily through didactics, self-learning, and hands-on patient care. Studies across the nation have highlighted inadequate training for house staff with regard to billing and coding.^3^ One report suggested that 82% of residents do not receive adequate training for billing and coding.^1^ Careful clinical documentation is essential not only for billing but also for communication, education, data collection, quality management, and research.^3,4^

Despite ongoing focus on education on billing and coding, studies have also shown that hospitals are at risk of incurring financial loss in revenue due to inaccurate billable documentation.^5^ Retrospective studies have indicated that the most specific International Classification of Diseases, Ninth Revision (ICD-9) codes were not often used and, instead, a nonspecific, miscellaneous code was, which posed a major limitation for retrospective data extraction.^6^ Cancer is a complex diagnosis, and studies have shown mistakes in accurate cancer documentation. One such publication identified more than 5,000 mistakes while reviewing over 26,000 tumor cases.^7^ With the implementation of ICD-10, oncological documentation has become more complex than ever. Several published papers have sought to improve documentation through tool kits, didactics, and personal feedback.^1,3–5,8–14^ Economic journals have also focused on medical documentation.^2^ Several studies have reported that educational sessions improve technical aspects of coding.^1,8^ Another publication demonstrated that personal feedback could improve aspects of resident learning.^9^ One curriculum utilized volunteer accrual and self-learning with self-evaluation as a means of education.^15^

We developed an educational tool kit that focuses on internal medicine residents and is based on hands-on learning, didactic sessions, and personal feedback to improve residents' oncologic documentation. Several *MedEdPORTAL* publications have addressed clinical documentation through didactics, testing, and small-group discussions.^16–22^ No current publication exists for combining didactics, hands-on learning, and personal feedback as an effective teaching tool in a 1-hour session. This curriculum focuses on internal medicine resident documentation skills for the inpatient setting. The one other publication that has focused on this population used vastly different outcomes, including complication codes, mortality, and a median quarterly case mix index.^14^ We report several unique outcomes that assess the existing knowledge base and inquire into the effectiveness of the module itself. Examples include inquiry about knowledge of billing and how it affects reimbursement, as well as assessment of the curriculum's impact regarding effectiveness of the didactic and personalized feedback. This tool kit also includes a standardized evaluation and feedback form for outcomes after the curriculum, including number of problems, chronicity of problems, specificity of problems, rationale for workup, and number of diagnoses discovered. This recipe can be applied to many aspects of learning during medical education training. We believe the incorporation of didactics, active learning, and personal feedback with a template for obtainable unique outcomes can be a very effective tool in this aspect of medical education.

## Methods

We created an educational curriculum at the Department of Medicine at the University of Florida in Gainesville. The curriculum was registered and approved through an institutional quality database, the Quality Improvement Patient Registry, and the Internal Medicine Residency Program. All the authors of this publication, as well as a representative from the billing department, met 10 times to brainstorm and create the curriculum. Each meeting was between 1 and 3 hours long, and the time was used to create, draft, and edit the mock histories and physicals (H&Ps), documentation PowerPoint, rubric, and pre/post surveys. No pretesting or cognitive interviewing prior to establishing the documentation was done; we felt the combination of two medical residents, a medical fellow, a representative from billing, and an experienced attending, along with several reviews, was adequate in creating the tool kit. We are willing to change any aspect of the tool kit to refresh and relaunch the experience for the next batch of internal medicine residents. Residents were recruited at a regularly scheduled didactic conference during a 1-hour session. A total of 24 residents participated in the curriculum. Nine postgraduate year (PGY) 1s, two PGY 2s, and three PGY 3s completed the full curriculum. As this was a didactic session replacing an educational noon conference, only individuals present that day for the educational conference were included. We chose not to repeat this lecture on additional days to avoid overexposure of the content to residents who had already participated. We also excluded participants if they did not complete both the preintervention and postintervention surveys. We utilized a conference room with a projector for the didactic and printed out copies of the surveys and mock H&P documentation.

First, a preintervention survey was handed out to establish a baseline of current awareness of comfort with clinical documentation for cancer patients (Appendix A). Thereafter, a mock H&P was distributed (Appendix B). The mock H&P, which we developed and reviewed with the billing department, was prefilled with history of present illness, review of systems, past medical and surgical history, family history, allergies, medications, vital signs, physical exam, labs, and imaging study results. Curriculum participants had to create their own assessment and plan based on the above information. We felt that with participants' allotted time constraints, rendering an assessment and plan would yield greatest benefits in terms of billing and documentation. After 20 minutes, a 20-minute didactic lecture was given during the same session (Appendix C). The lecture was given by a hematology/oncology fellow, and the PowerPoint used was created with the help of the university's billing department. The didactic session provided the basis for billing and coding for beginners. It included education on tips to complete a thorough and accurate assessment and plan. The lecture also focused on oncologic billing and nuances and specifics of ICD-10. Then, a second mock H&P was handed out (Appendix D). Both H&Ps had different characteristics and diagnoses but similar medical complexity and number of diagnoses. At the end of the 1-hour session, the presurvey and both mock H&Ps were collected. We scored the mock H&Ps utilizing a rubric (Appendix E). Both the pre- and the postintervention mock H&Ps were returned to the participants either in person or via electronic mail along with personalized suggestions for improvement and identified areas of strength. A postintervention survey was also provided to the participants at the end of the session to assess overall satisfaction with the educational tool kit and comfort with oncological documentation (Appendix F). Also included in this resource are standard H&Ps for comparison (H&P 1, Appendix G; H&P 2, Appendix H) and a summary of current studies addressing documentation (Appendix I).

## Results

**Figure 1.**
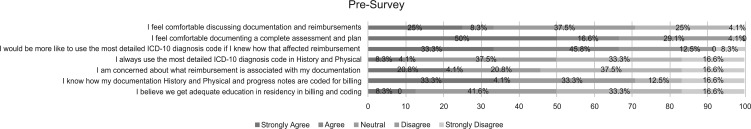
Preintervention survey responses in percentages. Abbreviation: ICD-10, International Classification of Diseases, Tenth Revision.

**Figure 2.**
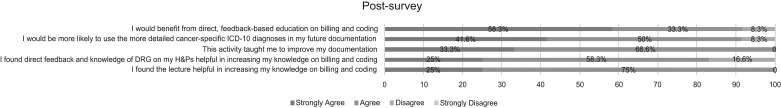
Postintervention survey responses in percentages. Abbreviations: DRG, diagnosis-related group; H&P, history and physical; ICD-10, International Classification of Diseases, Tenth Revision.

**Figure 3.**
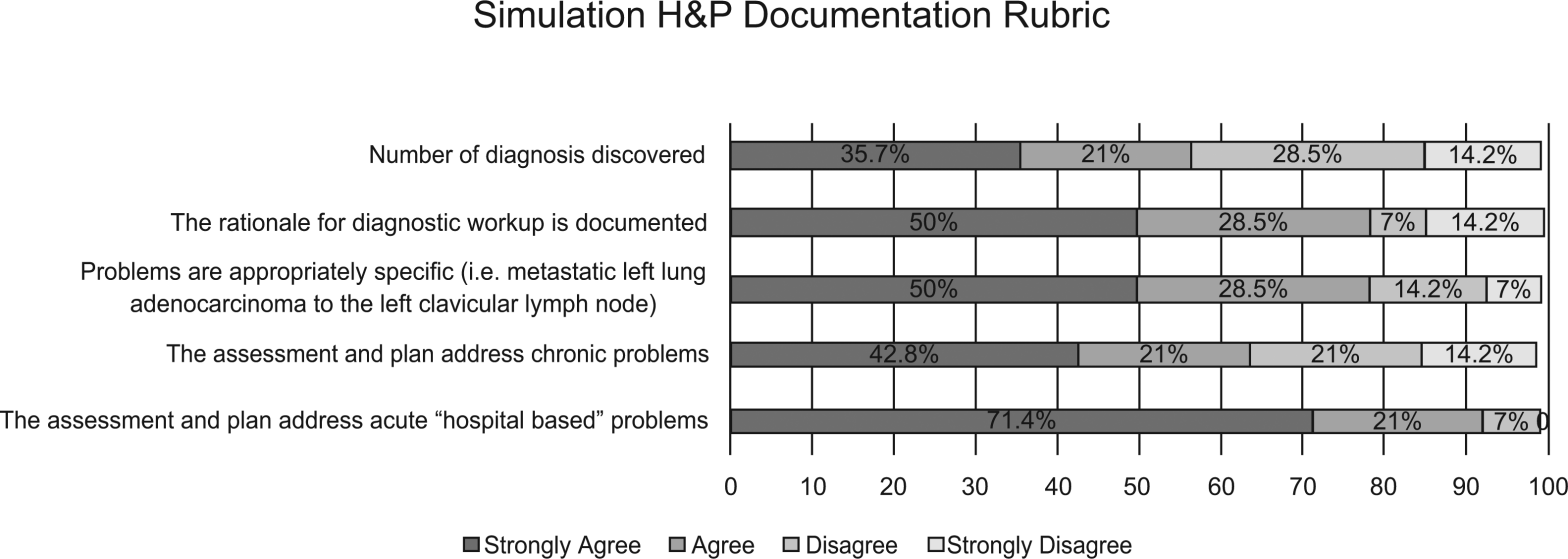
Compiled results from the standardized rubric assessing the second mock history and physical (H&P).

A total of 24 participants were recruited at the beginning of the curriculum for preintervention surveys, but only 12 participated in the postintervention survey. Figure 1 shows the preintervention survey responses, which were obtained as a baseline (*n* = 24). Postintervention survey results (*n* = 12) showed that 12 participants (100%) found this activity helpful, with three (25%) strongly agreeing and nine (75%) agreeing. Ten participants (83%) said that learning about diagnosis-related groups improved their knowledge of billing and coding, with three (25%) strongly agreeing and seven (58%) agreeing. All 12 curriculum participants (100%) felt that this activity taught them to improve documentation, with four (33%) strongly agreeing and eight (67%) agreeing. Eleven out of 12 participants (91%) felt they would be more likely to use cancer-specific ICD-10 diagnoses, with five (41%) strongly agreeing and six (50%) agreeing. Eleven out of 12 participants (91%) felt that they would benefit from direct feedback-based education, with seven (58%) strongly agreeing and four (33%) agreeing (Figure 2). Based on compiled results from the rubric, it appears residents had the most difficulty in addressing number of diagnoses discovered and assessing chronic medical problems (Figure 3).

## Discussion

Our educational intervention to teach accurate documentation for improved accuracy of coding and billing through the use of hands-on learning, didactics, and direct personalized feedback was well received by internal medicine residents. There was a high level of satisfaction with the intervention, with an average of 93% of residents noting a positive experience from the bundled educational module (including didactics and personalized feedback).

We recognize that there are limitations to these data. Our data stem from surveys, and our sample size is relatively small. We noticed a 50% dropout in survey responses from preintervention to postintervention questionnaires. This didactic and personalized feedback session may not significantly help those already possessing a good knowledge base of diagnosis-related groups and billing and coding, depending on individual strengths and academic level of training. When grading, we found it difficult to comprehend the written assessments and plans due to quick writing, scribbles, or just illegible writing. On occasion, we had to seek out individuals and ask them to clarify what they had written. However, the subjective positive response from 93% of our curriculum participants is certainly encouraging and shows that residents prefer more interactive, feedback-based education. Based on the data collected from the rubric, we feel medical residents have the best opportunity to improve their number of diagnoses and address chronical medical illness.

Throughout this educational endeavor, we learned that house staff have a difficult time engaging in an activity that may add to their overall workload. We also learned that constant reminders to complete a task are sometimes necessary if the task is not foremost on house staff's agenda. Future directions for this work may include a computer-based learning tool that allows users to complete the activity at their own discretion. This may also make it easier to grade the mock H&Ps as we are now in an age where handwritten notes are fading away and more users are accustomed to typing up documentation.

We believe our tool kit to improve documentation combines the most effective techniques to ensure adequate education for our house staff. Our priority was to combine a hands-on learning experience with a didactic, and consolidation of learning through personal feedback was the most effective way to ensure adequate training for billing and coding, an essential skill necessary for all physicians throughout their careers. Documentation is important for communication, efficiency, and billing. Education through hands-on learning, didactics, and personalized feedback provides a rigorous way to train house staff on optimal clinical documentation of cancer patients.

## Appendices

A. Preintervention Survey.docx
B. Blank H&P 1.docx
C. Billing and Coding Lecture.pptx
D. Blank H&P 2.docx
E. Standardized Rubric.docx
F. Postintervention Survey.docx
G. H&P 1.docx
H. H&P 2.docx
I. Summary of Current Studies.docx All appendices are peer reviewed as integral parts of the Original Publication.
