# Supplementary material for: An Interactive Multimodality Curriculum Teaching Medicine Residents About Oncologic Documentation and Billing
Source: MedEdPORTAL. 2018 Aug 30;14:10746. doi: 10.15766/mep_2374-8265.10746 (PMC6346345; doi:10.15766/mep_2374-8265.10746)
Supplement: Supplementary file 1 — A. Preintervention Survey.docx B. Blank H&P 1.docx C. Billing and Coding Lecture.pptx D. Blank H&P 2.docx E. Standardized Rubric.docx F. Postintervention Survey.docx G. H&P 1.docx H. H&P 2.docx I. Summary of Current Studies.docx [file mep-14-10746-s001.zip › A._Preintervention_Survey.docx]

| Table 1: Pre-survey Questionnaire | | |
| --- | --- | --- |
| Number of question | Question | Answer choices |
| 1 | I believe we get adequate education in residency in billing and coding | $⎕$ A) Strongly Agree  $⎕$ B) Agree  $⎕$ C) Disagree  $⎕$D) Strongly Disagree |
| 2 | I know how my documentation History and Physical and progress notes are coded for billing | $⎕$ A) Strongly Agree  $⎕$ B) Agree  $⎕$ C) Disagree  $⎕$D) Strongly Disagree |
| 3 | I am concerned about what reimbursement is associated with my documentation | $⎕$ A) Strongly Agree  $⎕$ B) Agree  $⎕$ C) Disagree  $⎕$D) Strongly Disagree |
| 4 | I always use the most detailed ICD-10 diagnosis code in History and Physical | $⎕$ A) Strongly Agree  $⎕$ B) Agree  $⎕$ C) Disagree  $⎕$D) Strongly Disagree |
| 5 | I would be more like to use the most detailed ICD-10 diagnosis code if I knew how that affected reimbursement | $⎕$ A) Strongly Agree  $⎕$ B) Agree  $⎕$ C) Disagree  $⎕$D) Strongly Disagree |
| 6. | I feel comfortable documenting a complete assessment and plan | $⎕$ A) Strongly Agree  $⎕$ B) Agree  $⎕$ C) Disagree  $⎕$D) Strongly Disagree |
| 7. | I feel comfortable discussing documentation and reimbursements | $⎕$ A) Strongly Agree  $⎕$ B) Agree  $⎕$ C) Disagree  $⎕$D) Strongly Disagree |
