# Supplementary material for: An Interactive Multimodality Curriculum Teaching Medicine Residents About Oncologic Documentation and Billing
Source: MedEdPORTAL. 2018 Aug 30;14:10746. doi: 10.15766/mep_2374-8265.10746 (PMC6346345; doi:10.15766/mep_2374-8265.10746)
Supplement: Supplementary file 1 — A. Preintervention Survey.docx B. Blank H&P 1.docx C. Billing and Coding Lecture.pptx D. Blank H&P 2.docx E. Standardized Rubric.docx F. Postintervention Survey.docx G. H&P 1.docx H. H&P 2.docx I. Summary of Current Studies.docx [file mep-14-10746-s001.zip › C._Billing_and_Coding_Lecture.pptx]

## Slide 1
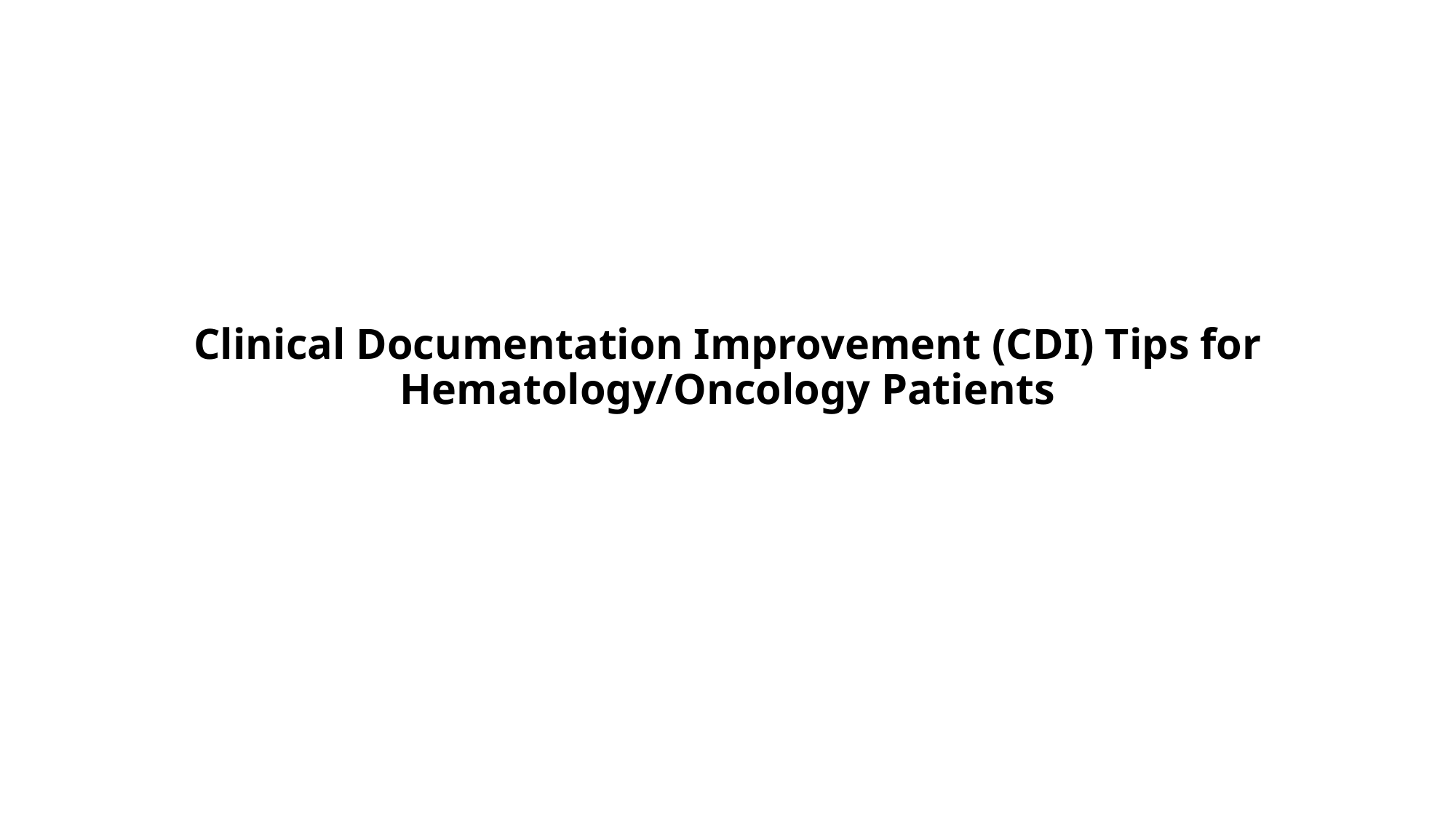

# Clinical Documentation Improvement (CDI) Tips for Hematology/Oncology Patients

## Slide 2
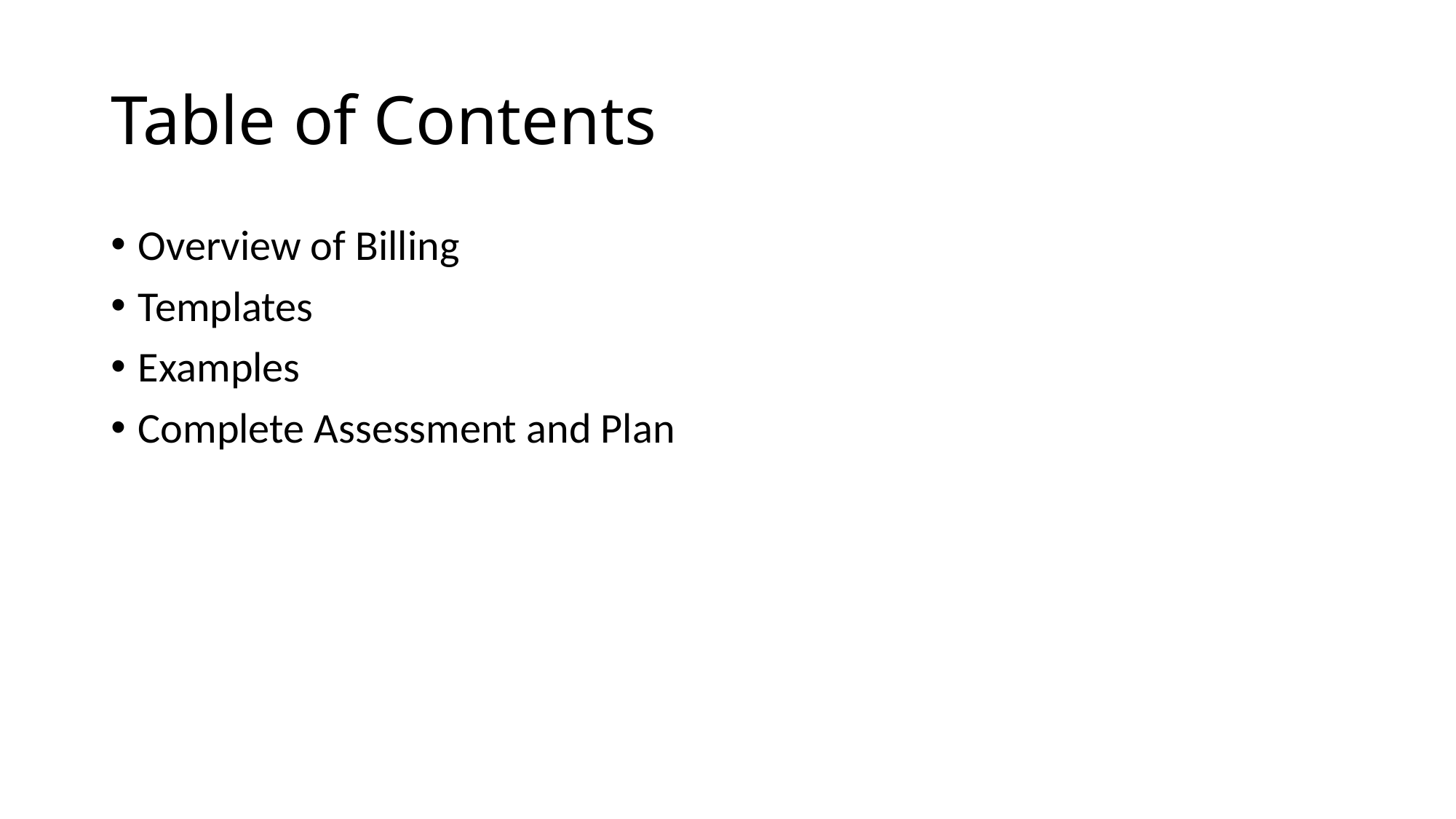

# Table of Contents
Overview of Billing
Templates
Examples
Complete Assessment and Plan

## Slide 3
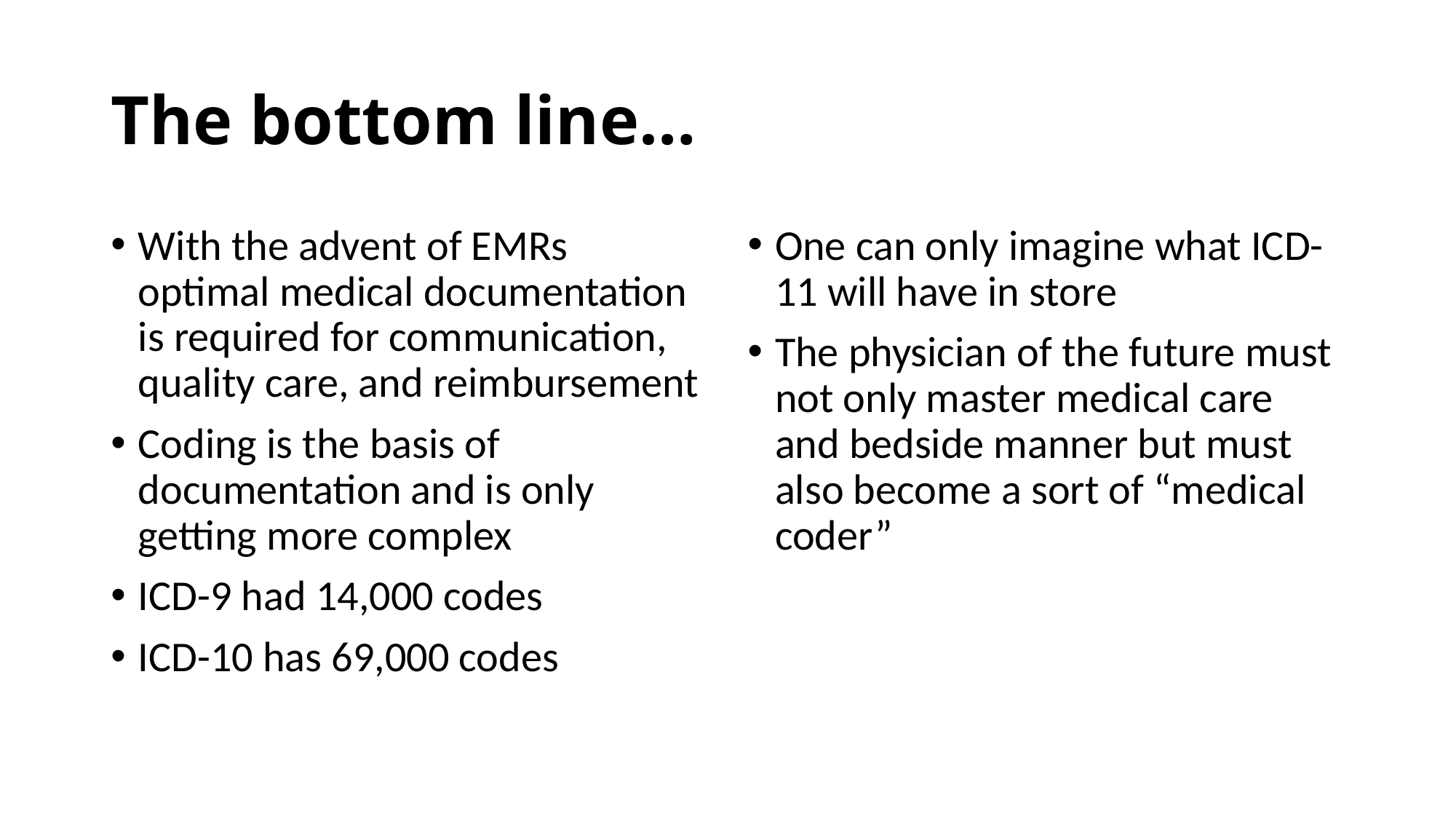

# The bottom line…
With the advent of EMRs optimal medical documentation is required for communication, quality care, and reimbursement
Coding is the basis of documentation and is only getting more complex
ICD-9 had 14,000 codes
ICD-10 has 69,000 codes
One can only imagine what ICD-11 will have in store
The physician of the future must not only master medical care and bedside manner but must also become a sort of “medical coder”

## Slide 4
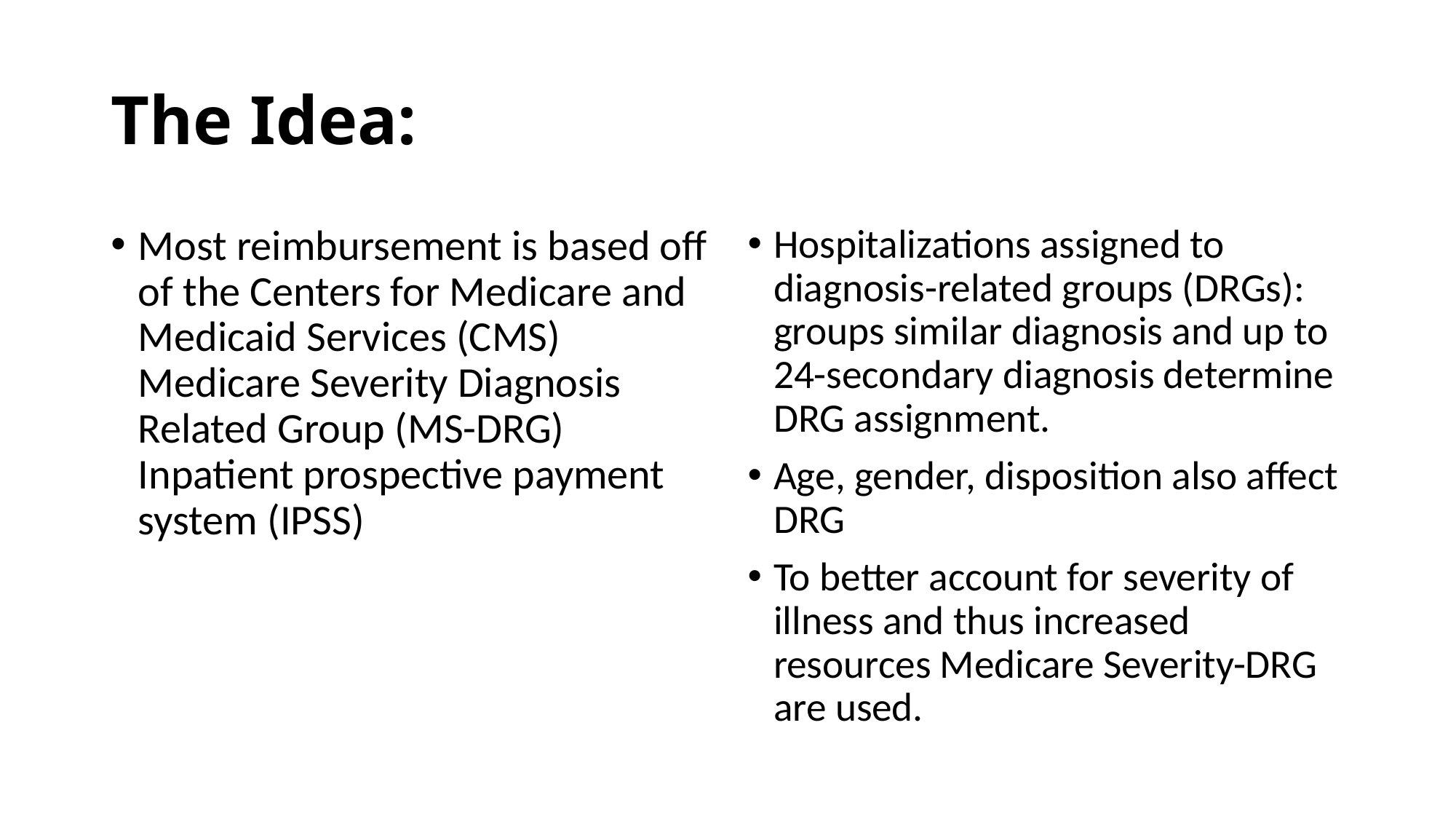

# The Idea:
Most reimbursement is based off of the Centers for Medicare and Medicaid Services (CMS) Medicare Severity Diagnosis Related Group (MS-DRG) Inpatient prospective payment system (IPSS)
Hospitalizations assigned to diagnosis-related groups (DRGs): groups similar diagnosis and up to 24-secondary diagnosis determine DRG assignment.
Age, gender, disposition also affect DRG
To better account for severity of illness and thus increased resources Medicare Severity-DRG are used.

## Slide 5
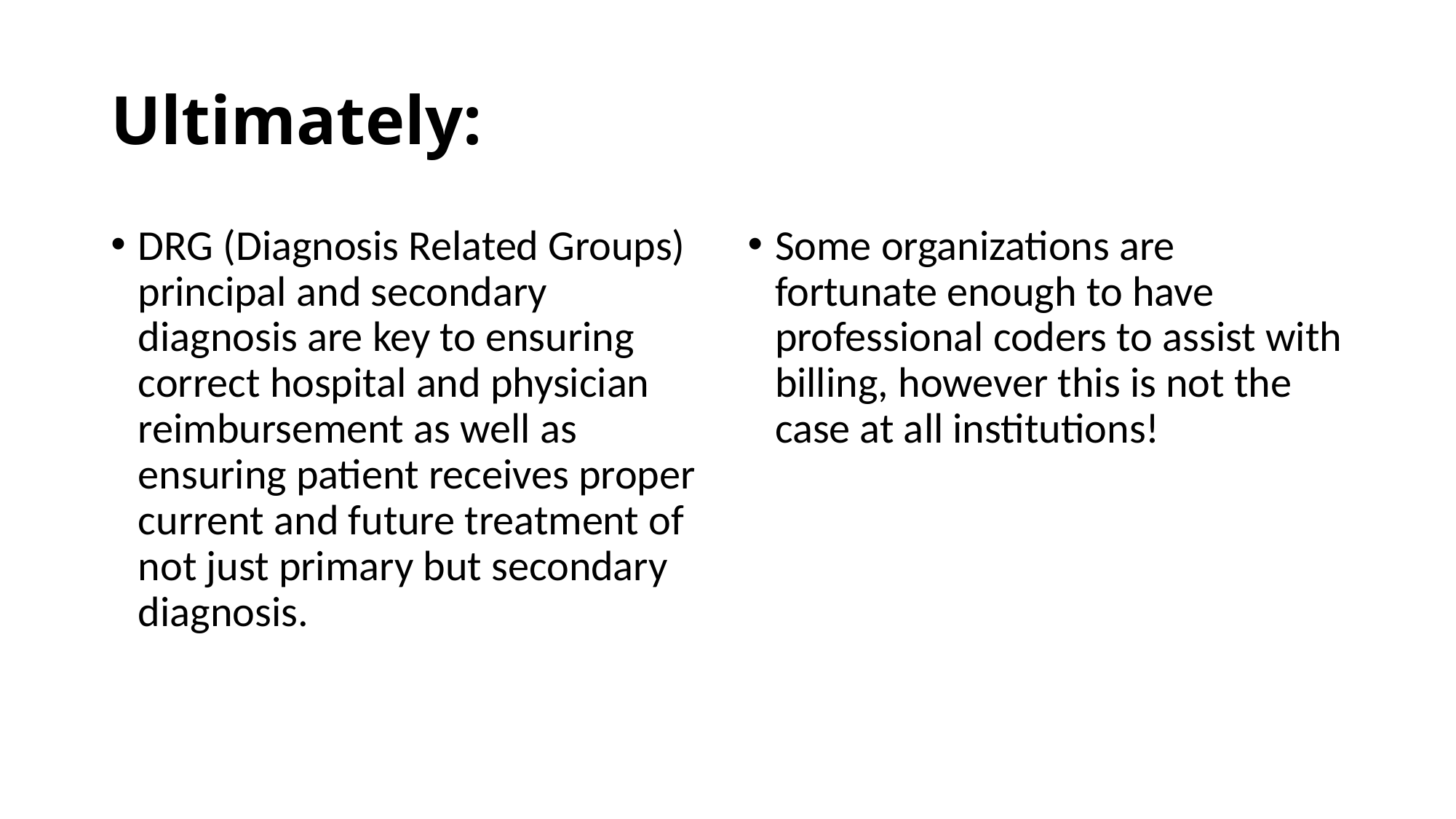

# Ultimately:
DRG (Diagnosis Related Groups) principal and secondary diagnosis are key to ensuring correct hospital and physician reimbursement as well as ensuring patient receives proper current and future treatment of not just primary but secondary diagnosis.
Some organizations are fortunate enough to have professional coders to assist with billing, however this is not the case at all institutions!

## Slide 6
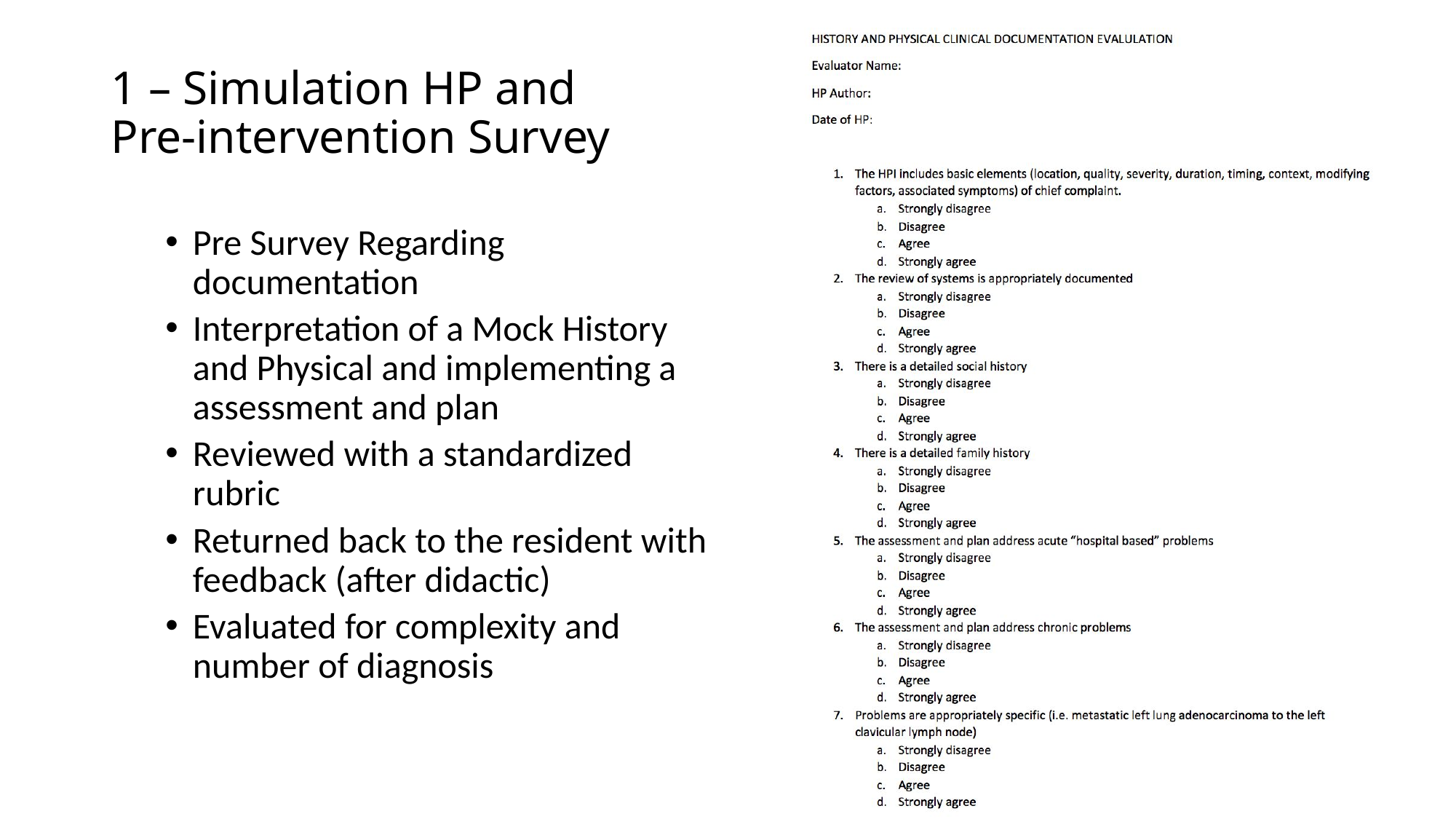

# 1 – Simulation HP and Pre-intervention Survey
Pre Survey Regarding documentation
Interpretation of a Mock History and Physical and implementing a assessment and plan
Reviewed with a standardized rubric
Returned back to the resident with feedback (after didactic)
Evaluated for complexity and number of diagnosis

## Slide 7
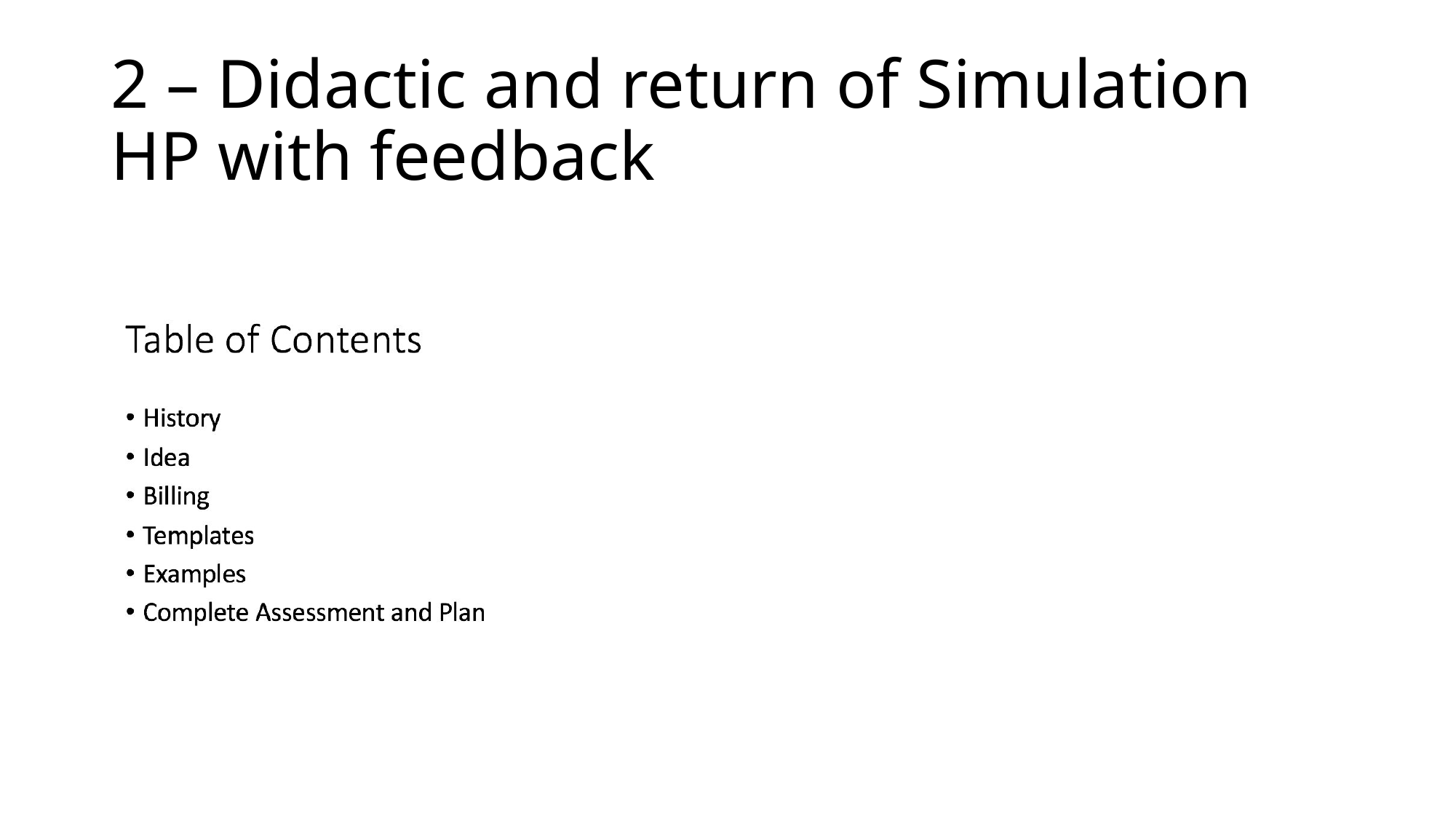

# 2 – Didactic and return of Simulation HP with feedback

## Slide 8
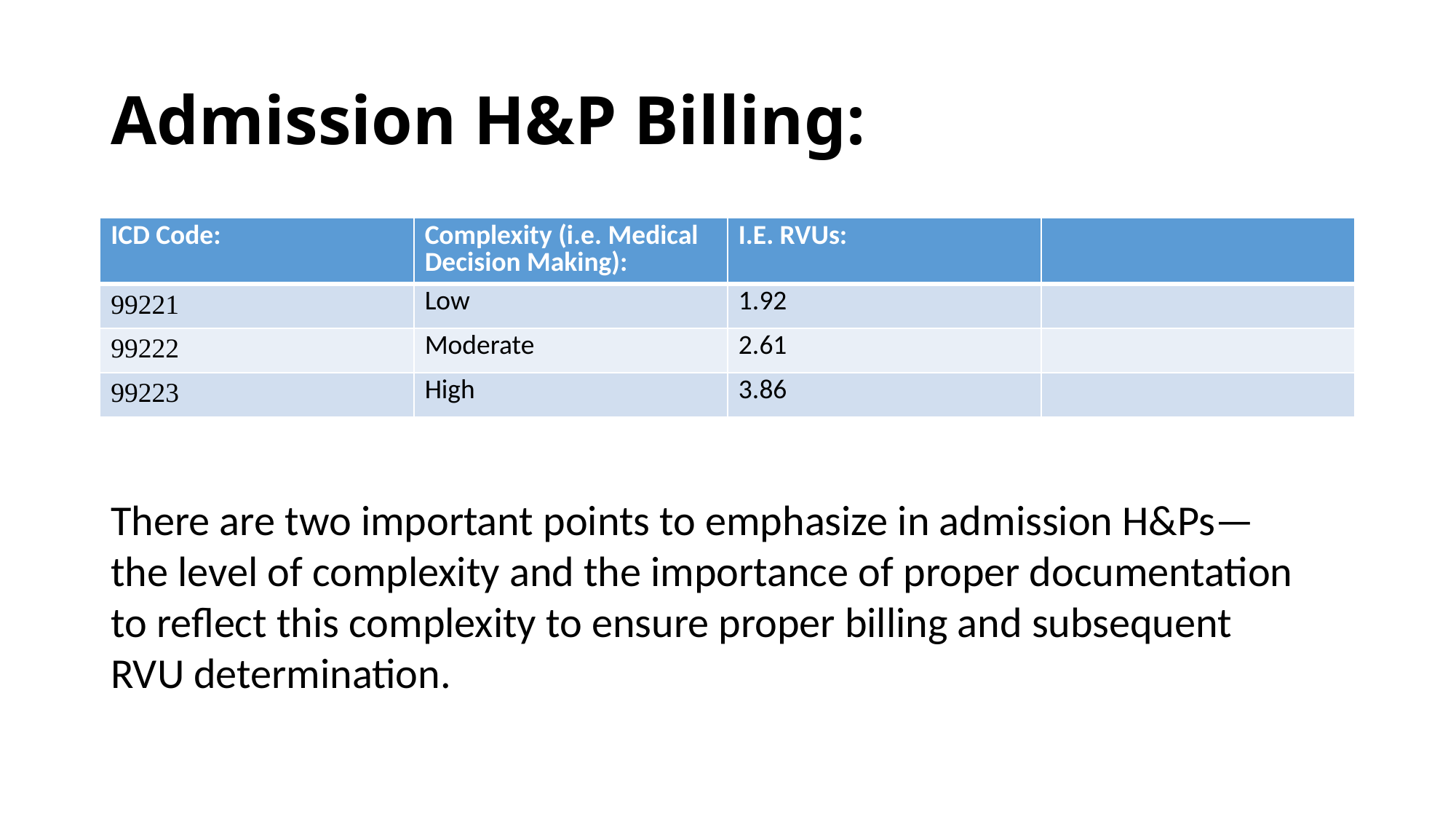

# Admission H&P Billing:
| ICD Code: | Complexity (i.e. Medical Decision Making): | I.E. RVUs: | |
| --- | --- | --- | --- |
| 99221 | Low | 1.92 | |
| 99222 | Moderate | 2.61 | |
| 99223 | High | 3.86 | |
There are two important points to emphasize in admission H&Ps—the level of complexity and the importance of proper documentation to reflect this complexity to ensure proper billing and subsequent RVU determination.

## Slide 9
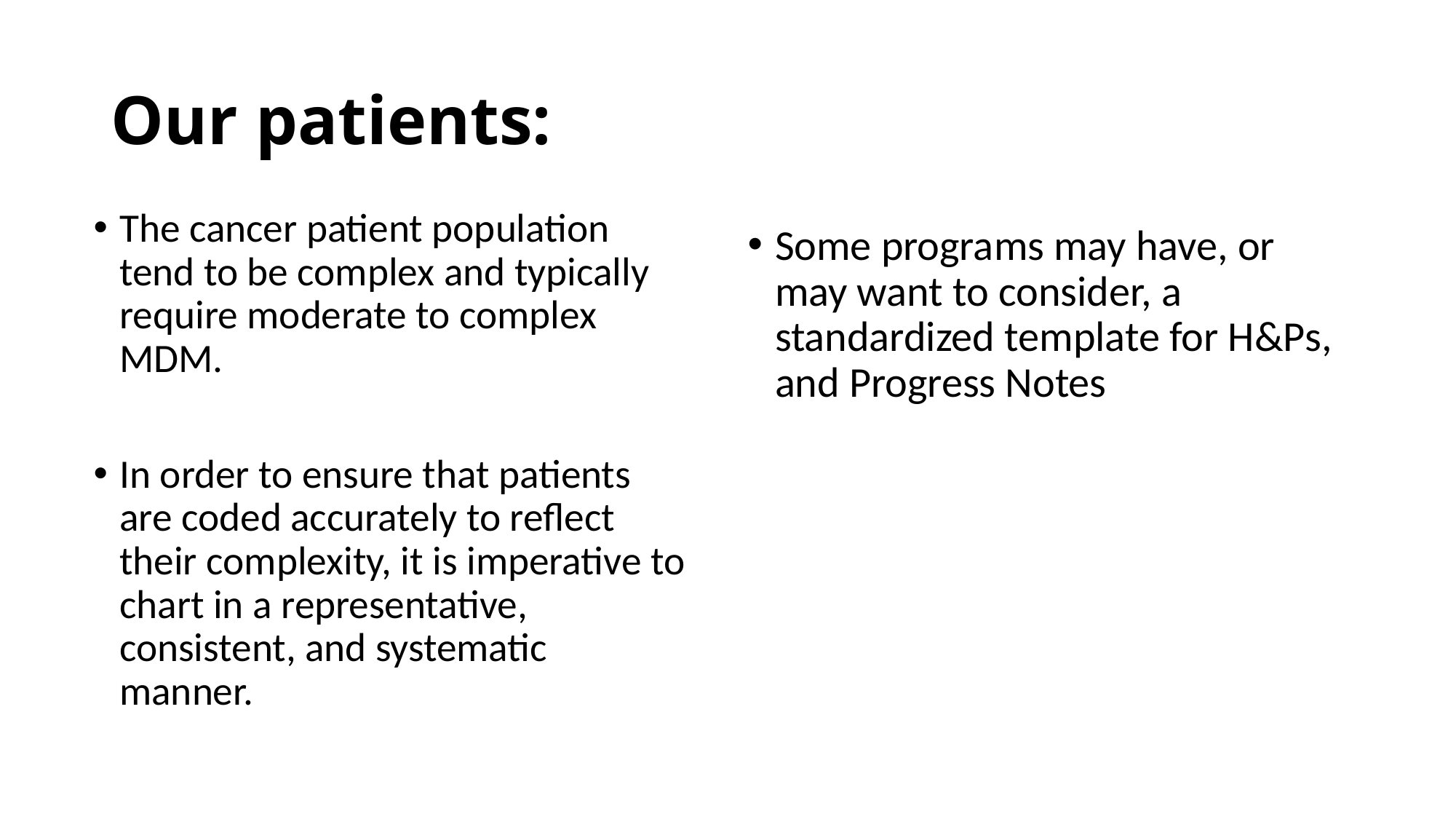

# Our patients:
The cancer patient population tend to be complex and typically require moderate to complex MDM.
In order to ensure that patients are coded accurately to reflect their complexity, it is imperative to chart in a representative, consistent, and systematic manner.
Some programs may have, or may want to consider, a standardized template for H&Ps, and Progress Notes

## Slide 10
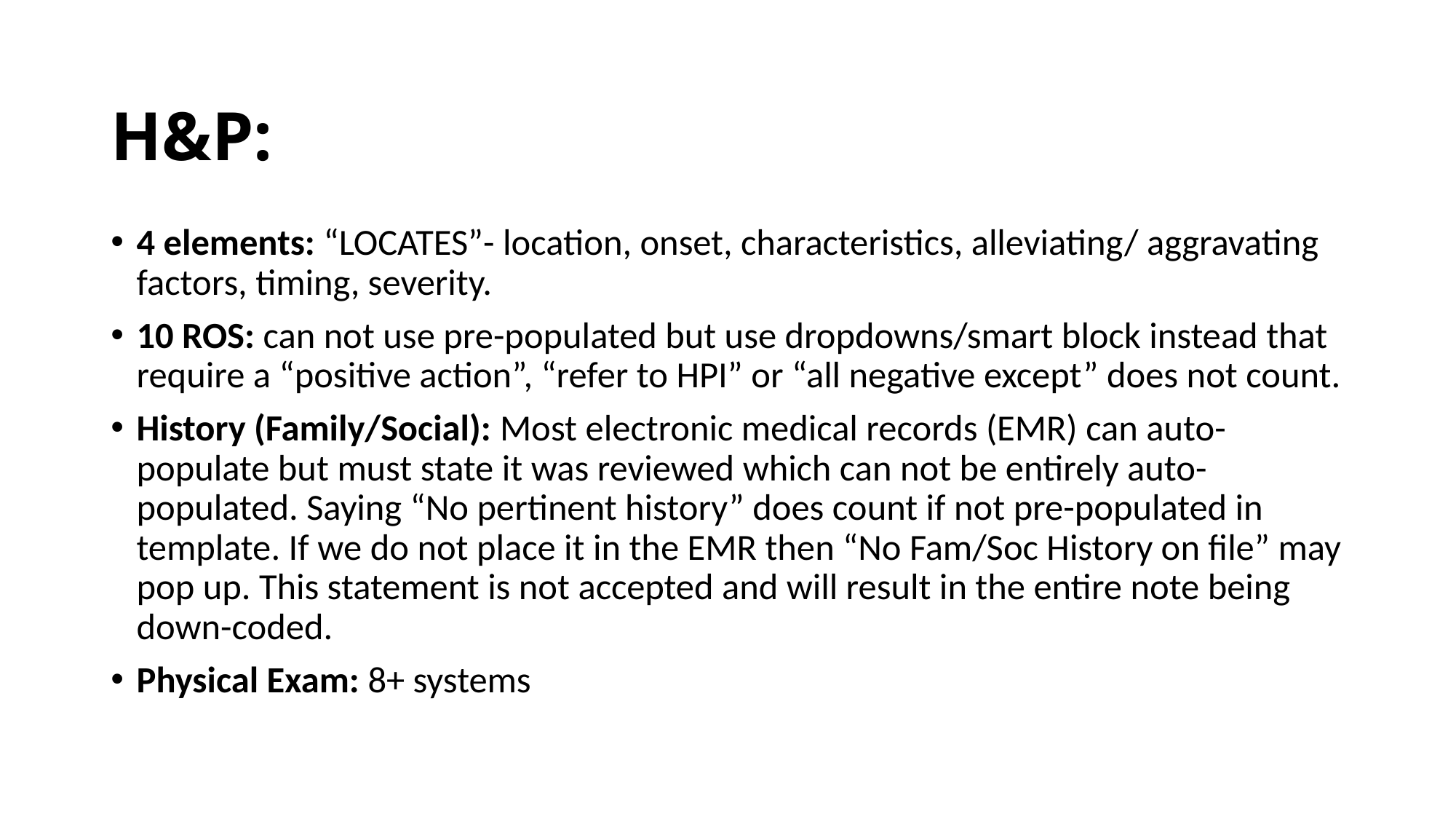

# H&P:
4 elements: “LOCATES”- location, onset, characteristics, alleviating/ aggravating factors, timing, severity.
10 ROS: can not use pre-populated but use dropdowns/smart block instead that require a “positive action”, “refer to HPI” or “all negative except” does not count.
History (Family/Social): Most electronic medical records (EMR) can auto-populate but must state it was reviewed which can not be entirely auto-populated. Saying “No pertinent history” does count if not pre-populated in template. If we do not place it in the EMR then “No Fam/Soc History on file” may pop up. This statement is not accepted and will result in the entire note being down-coded.
Physical Exam: 8+ systems

## Slide 11
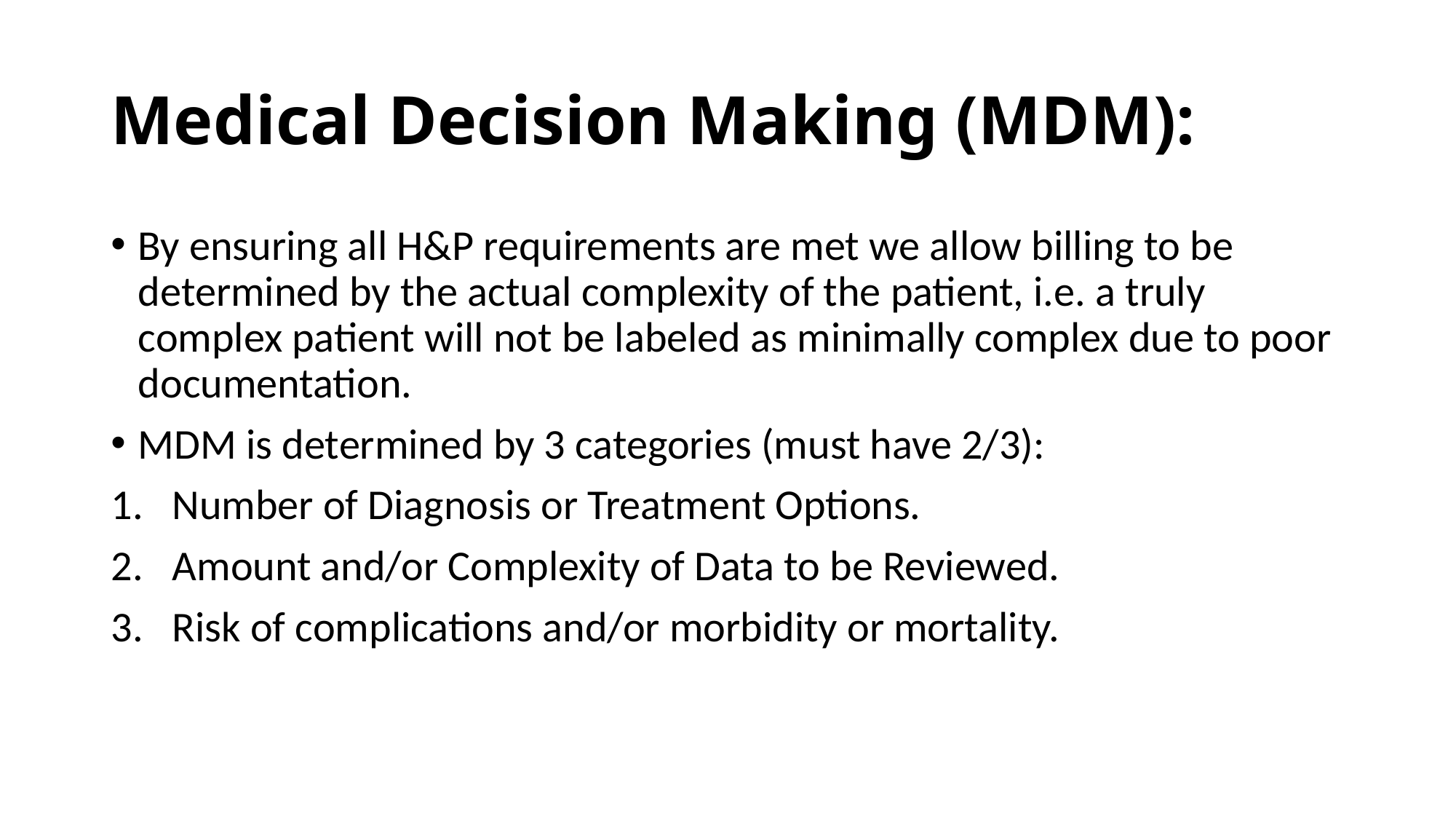

# Medical Decision Making (MDM):
By ensuring all H&P requirements are met we allow billing to be determined by the actual complexity of the patient, i.e. a truly complex patient will not be labeled as minimally complex due to poor documentation.
MDM is determined by 3 categories (must have 2/3):
Number of Diagnosis or Treatment Options.
Amount and/or Complexity of Data to be Reviewed.
Risk of complications and/or morbidity or mortality.

## Slide 12
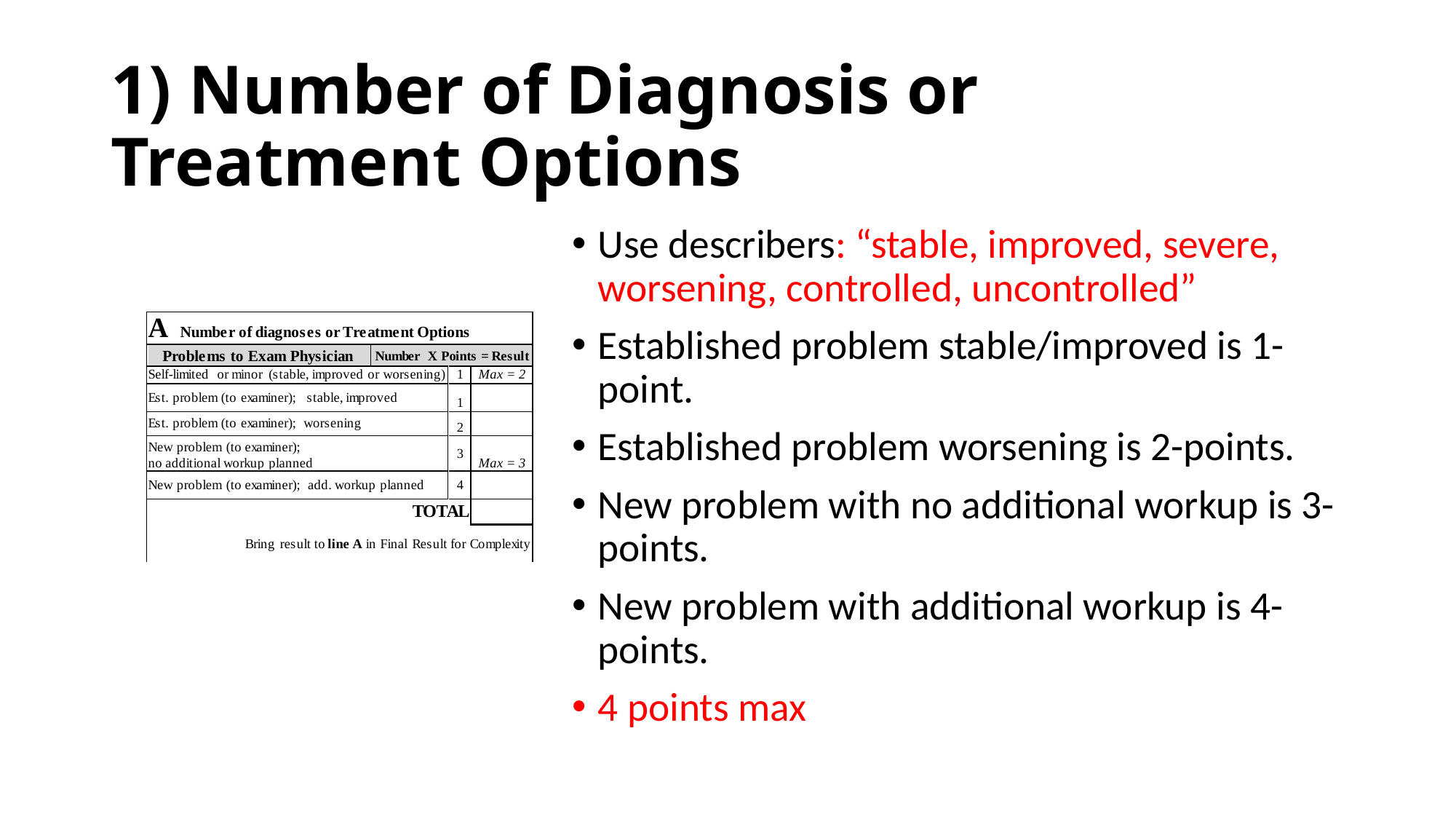

# 1) Number of Diagnosis or Treatment Options
Use describers: “stable, improved, severe, worsening, controlled, uncontrolled”
Established problem stable/improved is 1- point.
Established problem worsening is 2-points.
New problem with no additional workup is 3-points.
New problem with additional workup is 4-points.
4 points max

## Slide 13
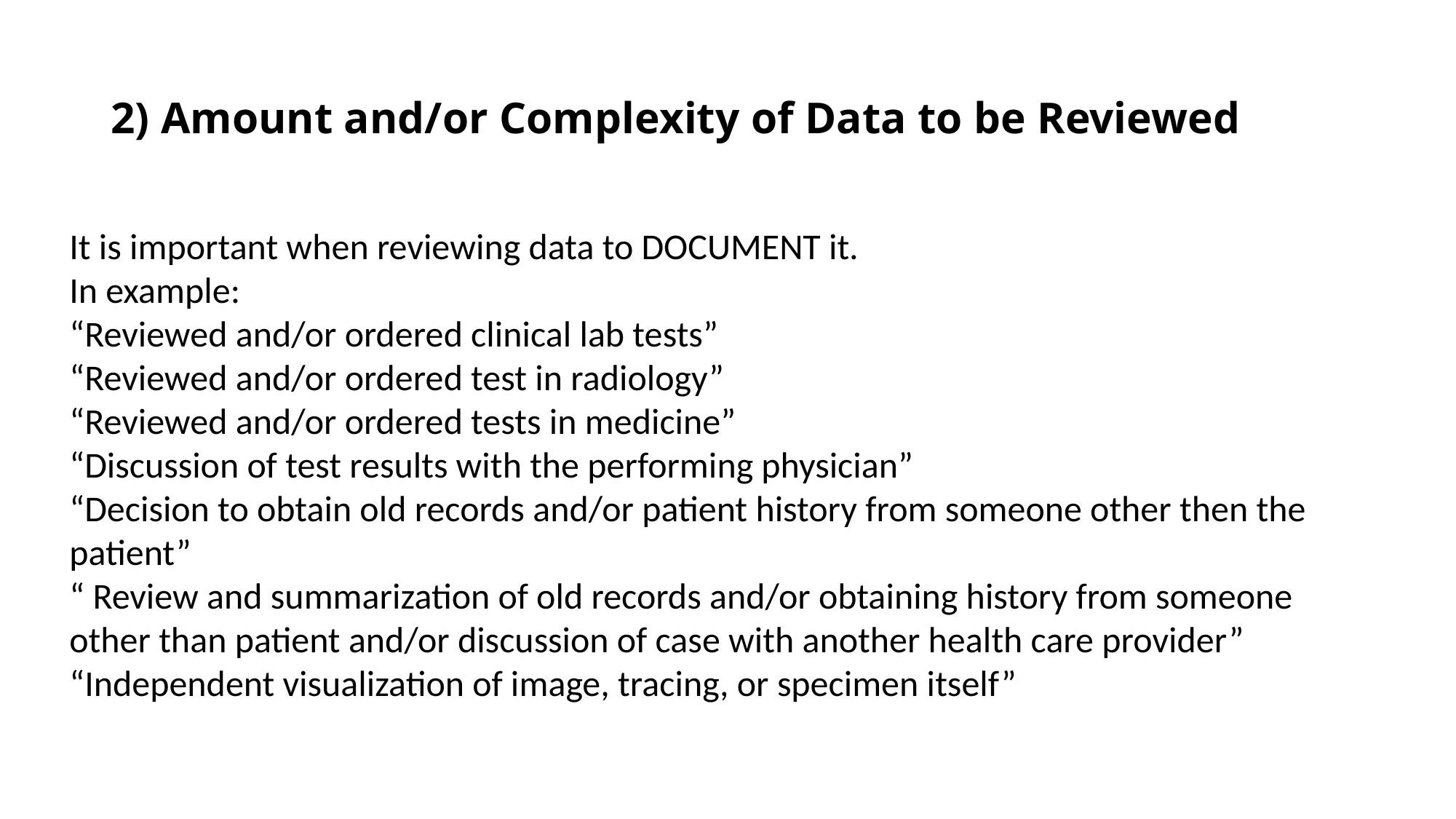

# 2) Amount and/or Complexity of Data to be Reviewed
It is important when reviewing data to DOCUMENT it.
In example:
“Reviewed and/or ordered clinical lab tests”
“Reviewed and/or ordered test in radiology”
“Reviewed and/or ordered tests in medicine”
“Discussion of test results with the performing physician”
“Decision to obtain old records and/or patient history from someone other then the patient”
“ Review and summarization of old records and/or obtaining history from someone other than patient and/or discussion of case with another health care provider”
“Independent visualization of image, tracing, or specimen itself”

## Slide 14
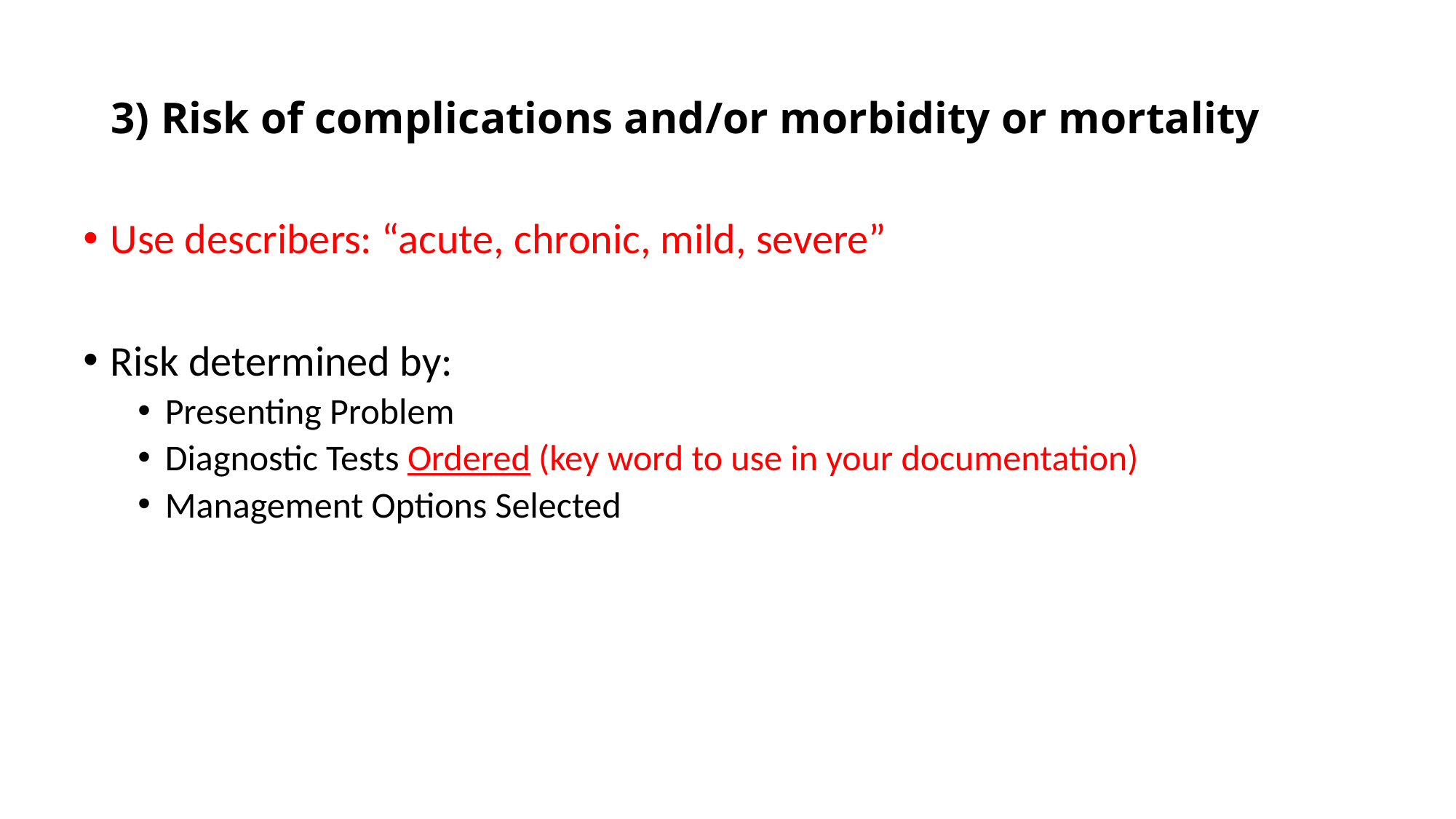

# 3) Risk of complications and/or morbidity or mortality
Use describers: “acute, chronic, mild, severe”
Risk determined by:
Presenting Problem
Diagnostic Tests Ordered (key word to use in your documentation)
Management Options Selected

## Slide 15
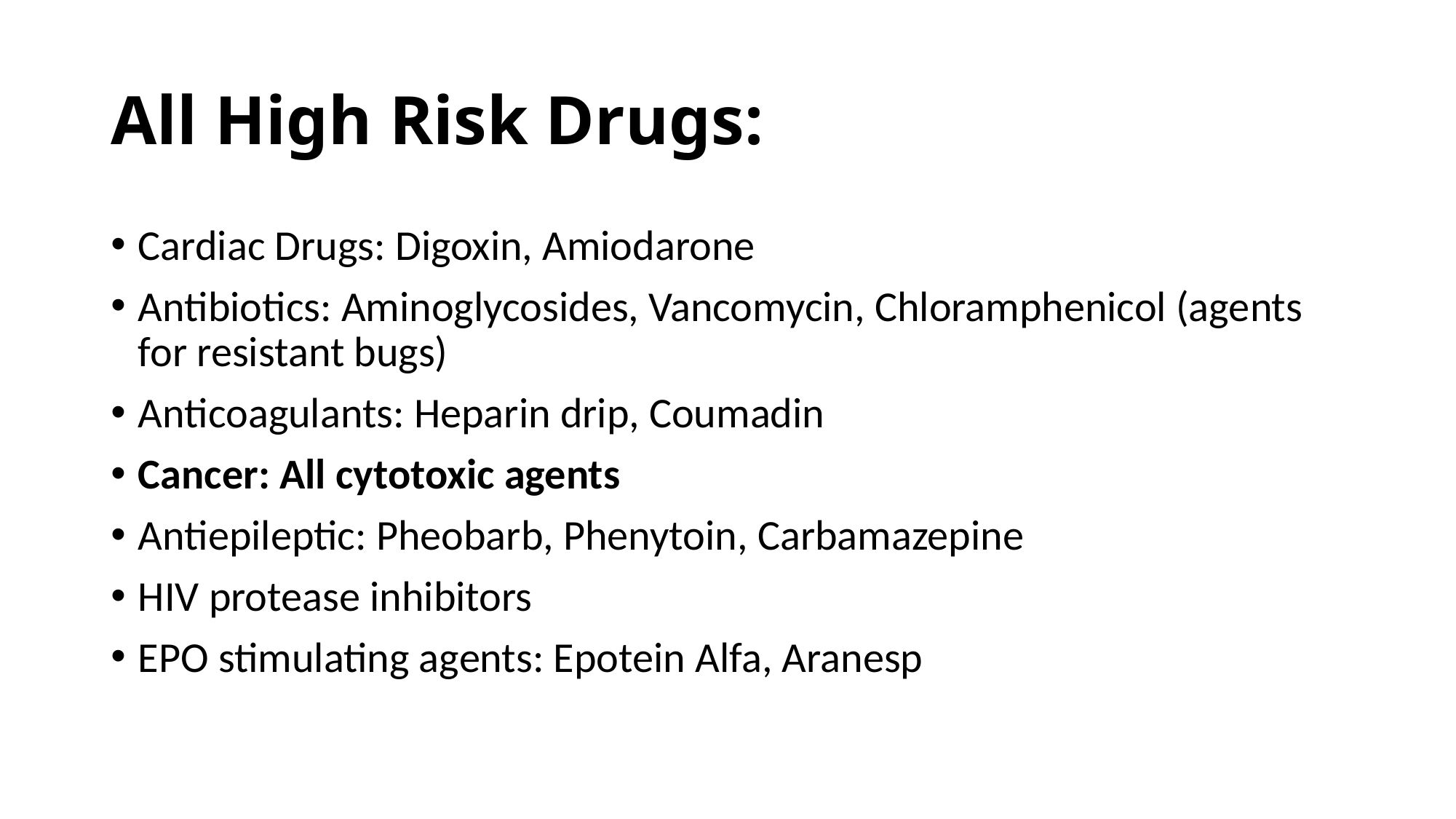

# All High Risk Drugs:
Cardiac Drugs: Digoxin, Amiodarone
Antibiotics: Aminoglycosides, Vancomycin, Chloramphenicol (agents for resistant bugs)
Anticoagulants: Heparin drip, Coumadin
Cancer: All cytotoxic agents
Antiepileptic: Pheobarb, Phenytoin, Carbamazepine
HIV protease inhibitors
EPO stimulating agents: Epotein Alfa, Aranesp

## Slide 16
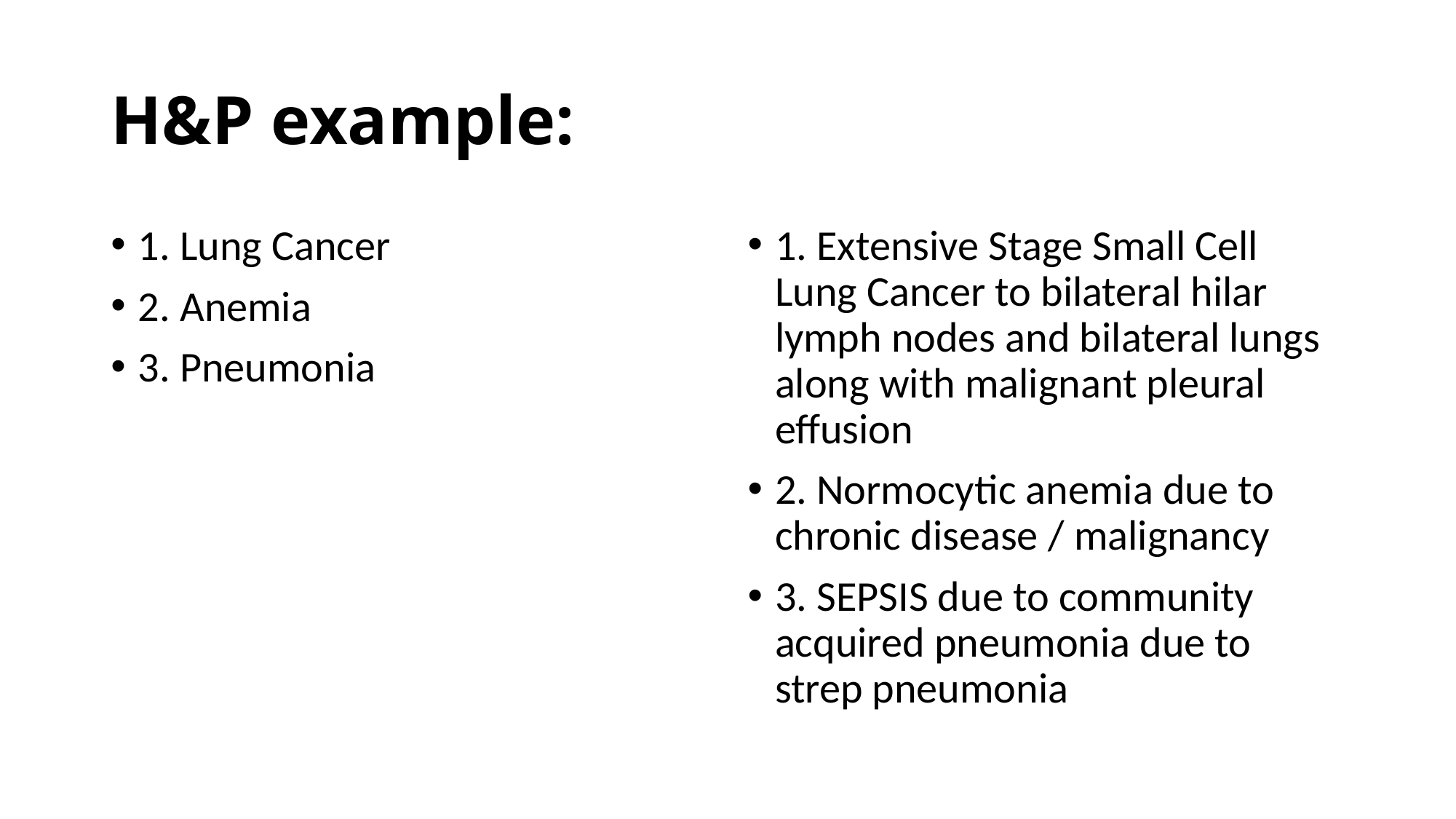

# H&P example:
1. Lung Cancer
2. Anemia
3. Pneumonia
1. Extensive Stage Small Cell Lung Cancer to bilateral hilar lymph nodes and bilateral lungs along with malignant pleural effusion
2. Normocytic anemia due to chronic disease / malignancy
3. SEPSIS due to community acquired pneumonia due to strep pneumonia

## Slide 17
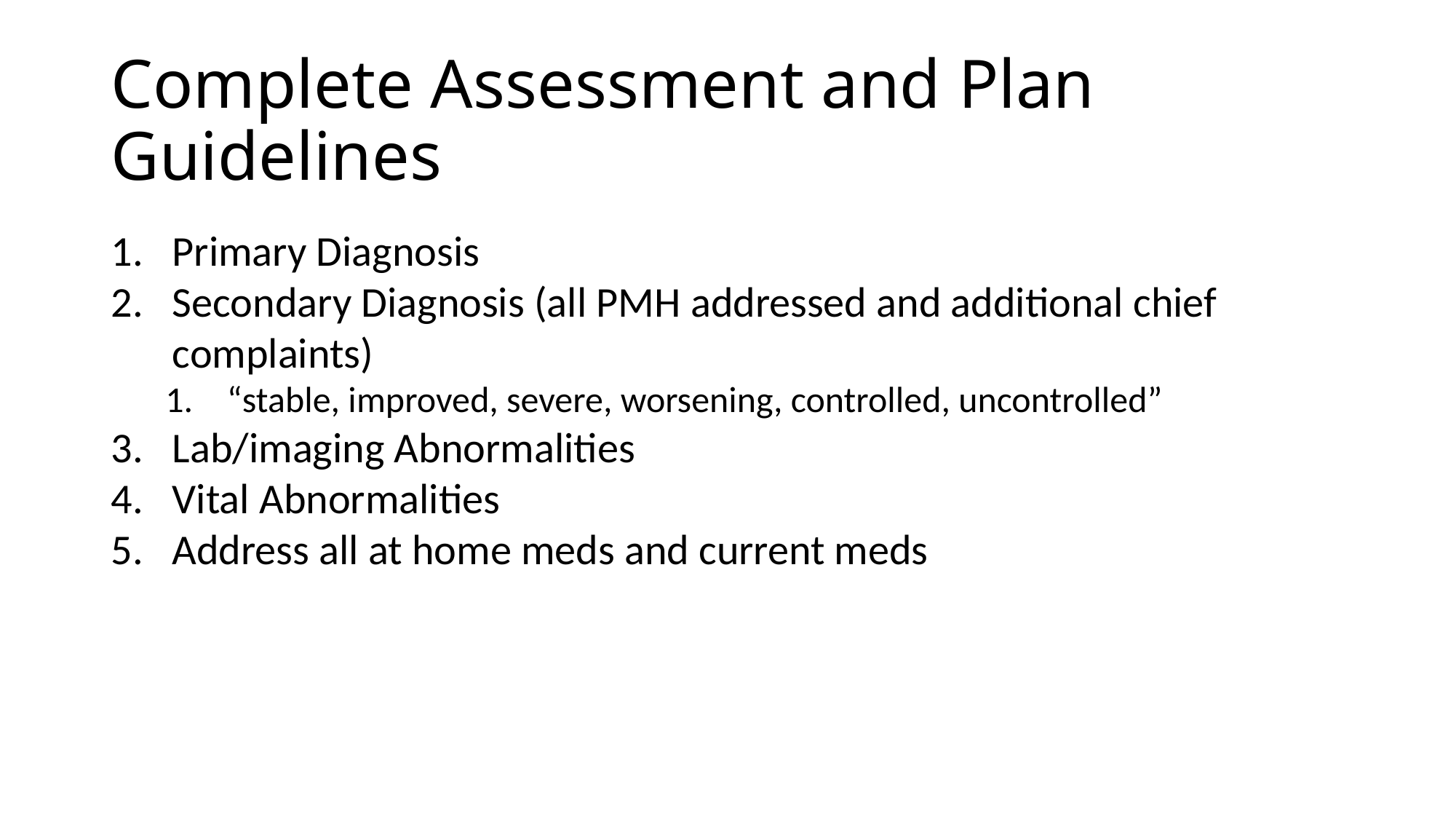

# Complete Assessment and Plan Guidelines
Primary Diagnosis
Secondary Diagnosis (all PMH addressed and additional chief complaints)
“stable, improved, severe, worsening, controlled, uncontrolled”
Lab/imaging Abnormalities
Vital Abnormalities
Address all at home meds and current meds

## Slide 18
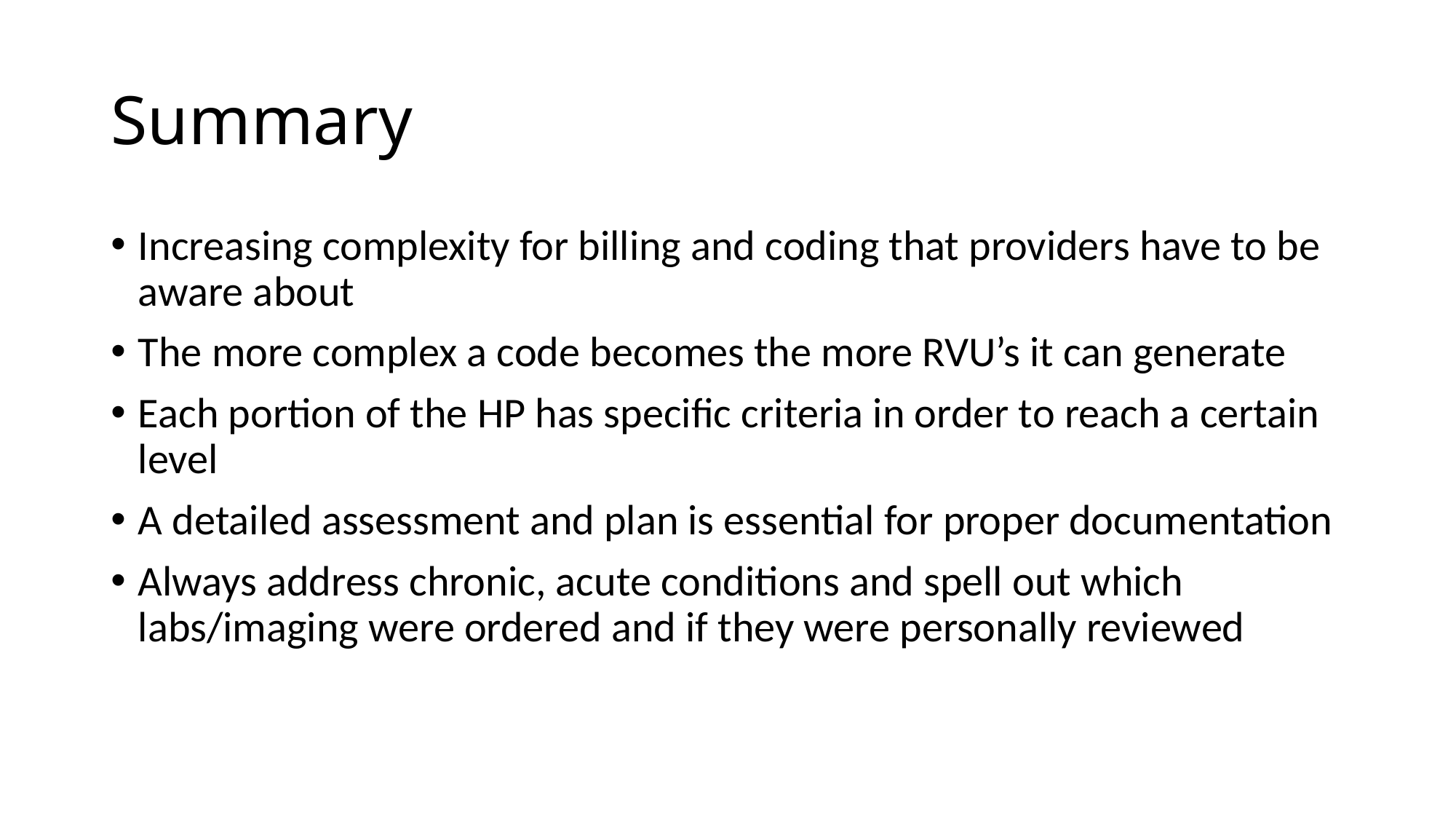

# Summary
Increasing complexity for billing and coding that providers have to be aware about
The more complex a code becomes the more RVU’s it can generate
Each portion of the HP has specific criteria in order to reach a certain level
A detailed assessment and plan is essential for proper documentation
Always address chronic, acute conditions and spell out which labs/imaging were ordered and if they were personally reviewed

## Slide 19
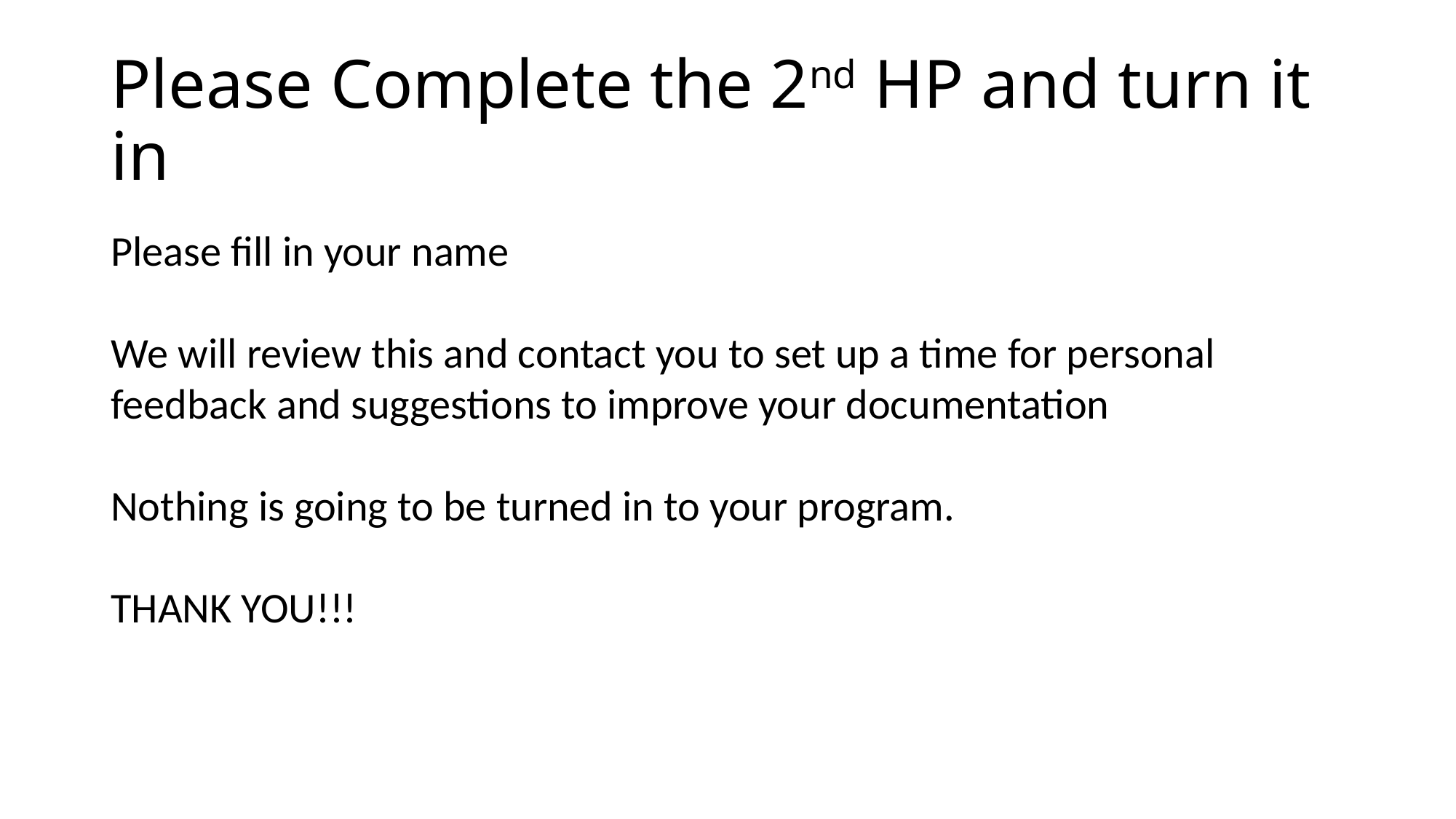

# Please Complete the 2nd HP and turn it in
Please fill in your name
We will review this and contact you to set up a time for personal feedback and suggestions to improve your documentation
Nothing is going to be turned in to your program.
THANK YOU!!!
