# Supplementary material for: An Interactive Multimodality Curriculum Teaching Medicine Residents About Oncologic Documentation and Billing
Source: MedEdPORTAL. 2018 Aug 30;14:10746. doi: 10.15766/mep_2374-8265.10746 (PMC6346345; doi:10.15766/mep_2374-8265.10746)
Supplement: Supplementary file 1 — A. Preintervention Survey.docx B. Blank H&P 1.docx C. Billing and Coding Lecture.pptx D. Blank H&P 2.docx E. Standardized Rubric.docx F. Postintervention Survey.docx G. H&P 1.docx H. H&P 2.docx I. Summary of Current Studies.docx [file mep-14-10746-s001.zip › E._Standardized_Rubric.docx]

SIMULATED HISTORY AND PHYSICAL CLINICAL DOCUMENTATION EVALULATION

Evaluator Name:

HP Author:

Date of HP:

1. The assessment and plan address acute “hospital based” problems
   1. Strongly disagree
   2. Disagree
   3. Agree
   4. Strongly agree
2. The assessment and plan address chronic problems
   1. Strongly disagree
   2. Disagree
   3. Agree
   4. Strongly agree
3. Problems are appropriately specific (i.e. metastatic left lung adenocarcinoma to the left clavicular lymph node)
   1. Strongly disagree
   2. Disagree
   3. Agree
   4. Strongly agree
4. There is an appropriate differential diagnosis in the assessment and plan
   1. Strongly disagree
   2. Disagree
   3. Agree
   4. Strongly agree
5. The rationale for diagnostic workup is documented
   1. Strongly disagree
   2. Disagree
   3. Agree
6. Number of diagnosis discovered
   1. Strongly disagree
   2. Disagree
   3. Agree
   4. Strongly agree
7. Comments:
