# Supplementary material for: An Interactive Multimodality Curriculum Teaching Medicine Residents About Oncologic Documentation and Billing
Source: MedEdPORTAL. 2018 Aug 30;14:10746. doi: 10.15766/mep_2374-8265.10746 (PMC6346345; doi:10.15766/mep_2374-8265.10746)
Supplement: Supplementary file 1 — A. Preintervention Survey.docx B. Blank H&P 1.docx C. Billing and Coding Lecture.pptx D. Blank H&P 2.docx E. Standardized Rubric.docx F. Postintervention Survey.docx G. H&P 1.docx H. H&P 2.docx I. Summary of Current Studies.docx [file mep-14-10746-s001.zip › G._H&P_1.docx]

**NAME:_____________________**

**Year (circle one)**

**PGY1 PGY2 PGY3**

**Department of Internal Medicine**

**History and Physical**

**CDI Team 1**

Admission Date and Time: 8/22/2017 4:15 PM

Primary Care Physician: Dr Adams Lopez

**CC:** shortness of breath

| **History of Present Illness:** |
| --- |

Mr. John Doe is a 67 y.o. male with a PMH significant for diabetes on metformin, anxiety controlled on medications, atrial fibrillation not on anticoagulation (personal choice), GERD on PPI, tobacco abuse who presents for dyspnea for one month. Patient states in the past month he has experienced 15lbs of weight loss, shortness of breath starting with exertion and now at rest, mild hemoptysis, and overall feeling unwell. He has not felt these symptoms in the past before. Symptoms started progressively. He has not had dyspnea prior to the last one month. He has a baseline cough but in the past few weeks noticed scant hemoptysis with coughing into a napkin. Mr Doe denies any sick contacts and recent travel. His dyspnea started slowly at rest and now he can only walk to his mailbox, which is 50 feet away from the front door. Denies any pain. States he has slight nausea without any vomiting. Regarding his cough – he has a baseline cough for the past several months without any green or yellow phlegm. Since the past few weeks he has been noticing green discharge along with streaks of blood in the phlegm. Associated symptoms are fevers, chills, and occasional night sweats where he has to change his clothes in the morning. Regarding his weight loss – patient’s diet has not changed, complaints of slight decrease appetite, and the weight loss is unintentional. He has had stable weights for several years until this point. Denies any neurological complaints at this time. Denies any chest pain along with the dyspnea, also denies any lower extremity swelling and pain. He has been compliant with his medications in the past several weeks without missing any doses. He also complains of fatigue and an overall unwell feeling. Please review ROS for more details.

| **Review of Systems:** |
| --- |

**ROS:**

Constitutional Symptoms: positive for unexplained weight loss, night sweats, fatigue/malaise/lethargy, decrease in appetite, fevers. Denies any sleep changes, itch/rash, recent trauma, lumps/bumps/masses, unexplained falls

Eyes: negative for visual changes, headache, eye pain, double vision

Ears, Nose, Mouth: positive for runny nose. Denies any epistaxis, sinus pain, stuffy ears, ear pain, tinnitus, gingival bleeding, toothache, sore throat, pain with swallowing

Cardiovascular: positive for occasional chest discomfort and/or palpitations and dyspnea, negative for lower extremity edema, loss of consciousness

Respiratory: positive for cough, sputum, hemoptysis, dyspnea, exercise intolerance

Gastrointestinal: positive for nausea, negative for abdominal pain, difficulty swallowing, indigestion, cramping, diarrhea, constipation, hematemesis, bright blood per rectum, foul smelling stools, melena

Genitourinary: negative for incontinence, dysuria, nocturia, polyuria, vomiting. Positive for nausea

Musculoskeletal: negative for pain, stiffness, joint swelling, decreased range of motion

Integumentary: negative for pruritus, rashes, stria, lesions, wounds, incisions, nodules, eczema

Neurological: negative for changes in sight, smell, hearing, taste, headaches, paraesthesiaes, numbness, limb weakness, poor balance, speech problems

Psychiatric: negative for depression, difficulty in concentration, paranoia, mania, changes in personality. Positive for anxiety.

Endocrine: Negative for prefer cold/hot weather, sweating, diarrhea, depression, dry skin, erection issues. Positive for polyuria.

Hematologic/Lymphatic: negative for purpura, petechial, history of bleeding issues

Allergic/Immunologic: negative for signs or symptoms of anaphylaxis, groin/axillary/neck pain/lumps/bumps, no environmental allergies

| **Past Medical and Surgical History:** |
| --- |

| **Past Medical History:** | | |
| --- | --- | --- |
| Diagnosis | | Date |
| • | Diabetes, A1C 7.6, with peripheral neuropathy | 5/2010 |
| • | Anxiety | 8/2016 |
| • | Atrial fibrillation | 2/2012 |
| • | GERD (gastroesophageal reflux disease) |  |

| **Past Surgical History:** | | | |
| --- | --- | --- | --- |
| Procedure | | Laterality | Date |
| • | CHOLECYSTECTOMY |  | 04/23/2013 |
| • | EYE SURGERY |  | 1999 |
| • | TONSILLECTOMY |  | 1990 |

| **Social History:** |
| --- |

| **Social History** |
| --- |

| Social History | | |
| --- | --- | --- |
| • | Marital status: | Single |
| • | Number of children: | 0 |

| Social History Main Topics | | | | | | | |
| --- | --- | --- | --- | --- | --- | --- | --- |
| • | Smoking status: | | | Current Every Day Smoker | | | |
|  |  | Packs/day: | | 2ppd for 47 years | | | |
|  |  | Last attempt to quit: | | Never | | | |
| • | Smokeless tobacco: | | | Never Used | | | |
| • | Alcohol use | | | Yes | | |  |
|  |  |  | *Comment: occasionally* | | | | |
| • | Drug use: | | | No | | | |
| • | Sexual activity: | | | No | | | |
|  |  | | |  | | | |
| Social History Narrative | | | | | |  |  |
| Patient lives alone in a 2 story house. He able to tend to all of his ADLs. Never married. Not currently sexually active. Patient is not up to date with his colonoscopy. He currently works as a construction worker operating heavy machinery. Denies any travel outside of the state in the past five years. Has a good strong social support circle from friends and family. | | | | |  |  |  |

| **Family History:** |
| --- |

| **Family History** | | | |
| --- | --- | --- | --- |
| Problem | | Relation | Age of Onset |
| • | Cancer | Father | 68 |
| • | Cancer | Brother | 52 |
| • | Arthritis | Mother |  |

I have reviewed the past medical, past surgical, family and social history.

| **Allergies:** |
| --- |

| **Allergies** | | |
| --- | --- | --- |
| Allergen | | Reactions |
| • | Penicillin G | Nausea Only |
| • | Erythromycin | Anxiety |

| **Home Medications:** |
| --- |

| **Prior to Admission medications** | | | | | |
| --- | --- | --- | --- | --- | --- |
| Medication | Sig | Start Date | End Date | Taking? | Authorizing Provider |
| **Metformin 1000mg XL** | **1 tablet po q BID** |  |  | **Yes** | **Information, Historical** |
| **ALPRAZolam (XANAX) 0.25 MG tablet** | **Take 1 Tablet by mouth BIDP as needed for anxiety** | **8/22/2016** |  |  | **Doc, McStuffins, M.D.** |
| **aspirin 81 MG Tablet** | **Take 81 mg by mouth daily.** |  |  |  | **Information, Historical** |
| **Inv esomeprazole (NexIUM) 40mg capsule** | **Take by mouth nightly at bedtime.** |  |  |  | **Information, Historical** |
| **metoprolol succinate (TOPROL-XL) 25 MG Tablet Extended Release 24 Hour** | **Take 1 tablet by mouth daily.** | **2012** |  |  | **Information, Historical** |

| **Objective:** |
| --- |

| **Vital Signs: Last Filed** | **Vitals Signs: 24 Hour Range** |
| --- | --- |
| Temp: 38.8 °C (101.84 °F) (08/22 1600) | Temp: [(37.8°C -38.8 °C)] |
| Pulse: 130 (08/22 1600) | Pulse: [71-160] |
| BP: 108/74 (08/22 1600) | BP: (101-168)/(60-116) |
| Resp: 29 (08/22 1600) | Resp: [16-35] |
| SpO2: 82 % (08/22 1600) | SpO2: [80 %-88 %] |

**Weight** **:**

| **Wt Readings from Last 3 Encounters:** | |
| --- | --- |
| 08/22/17 | 37.9 kg |

**Constitutional:** appears ill, calm, cooperative, appears in distress

**Eyes:** extra-ocular movements in-tact, no scleral icterus, PERRL

**Ears/Nose/Throat/Mouth/Neck:** dry mucous membranes without lesions, neck soft and supple

**Cardiovascular:** tachycardic rate, irregular rhythm, no murmur, rub or gallop, 2+ radial pulses, no pitting edema

**Respiratory:** tachypnea noted, clear breath sounds in upper lobes, slight crackles at the bases, using respiratory muscles to breathe

**Gastrointestinal:** +BS, abdomen soft, non-tender and non-distended

**Musculoskeletal:** no joint swelling or clubbing

**Skin:** normal turgor, no hyper/hypo pigmentation

**Neurological:** moves all extremities, no facial asymmetry, CNII-XII grossly intact, no focal deficits

**Psychiatric:** A&Ox3, normal mood and affect

**Access:** peripheral line

| **Data Review:** |
| --- |

I have reviewed the following laboratory studies:

**Recent Labs**

| **Lab** | **08/22/17 1700** |
| --- | --- |
| NA | 122 (136-145 mmol/L) |
| K | 3.6 (3.3-5.1 mmol/L) |
| CL | 101 (98-107 mmol/L) |
| CO2 | 26 (22-30 mmol/L) |
| BUN | 54 (6-21 mg/dL) |
| CREATININE | 2.5 (0.38-1.02 mg/dL) |
| GLU | 180* (65-99 mg/dL) |
| CALCIUM | 7.9* (8.4-10.2mg/dL) |
| MG | 2.4 (1.5-2.8 mg/dL) |
| PHOS | 2.9 (2.7-4.5 mg/dL) |

**Recent Labs**

| **Lab** | **08/22/17 1700** |
| --- | --- |
| TPROT | 5.8* (6.4-8.3 g/dL) |
| ALB | 2.3 (3.5-5.2 g/dL) |
| AST | 51 (0-37 IU/L) |
| ALT | 70 (0-35 IU/L) |
| TBILI | 0.8 (0.0-1.0 mg/dL) |
| DBILI | 0.2 (0.0-0.2 mg/dL) |
| ALKPHOS | 179 (40-150 IU/L) |

**Recent Labs**

| **Lab** | **08/22/17 1700** |
| --- | --- |
| PROTIME | 13.1 (9.1-13.5 seconds) |
| INR | 1.2* (0.8-1.1) |

**Recent Labs**

| **Lab** | **08/22/17 1700** |
| --- | --- |
| WBC | 15* (3.0-10.0 thou/cu mm) |
| HGB | 9 (12.0-16.0 g/dL) |
| HCT | 27 (35.0-45.0%) |
| PLATCOUNT | 250 (150-450 thou/cu mm) |

MCV 85 (78.0-100.0 fl)

Differential:

Neutrophilic shift as per manual review by technologist

| **Lab** | **08/22/17 1700** |
| --- | --- |
| HbA1c | 7.6 (4.1-6.1%) |
| **Lab** | **08/22/17 1700** |
| LDH | 500 (95-129 mg/dL) |
| Uric Acid | 7.4 (0.0-7.0 mg/dL) |

| **Lab Results** | | | |
| --- | --- | --- | --- |
| Component | | Value | Date/Time |
|  | TROPONINI | <0.03 (<0.04 ng/mL) | **08/22/17 1700** 11:00 AM |

**Imaging:**

**CXR** - I have reviewed the study dated 8/22/17 and it shows: ground glass opacities in bilateral lobes, significant pleural effusion in the right lower lobe, 6cm mass located in the right middle lobe

**CT Chest w/contrast -** I have reviewed the study dated 8/22/17 and it shows likely malignant lesion in the right lung that is 6.3cm x 5.3cm, associated adenopathy, and a significant pleural effusion in the right lower lobe. Appears to have post-obstructive pneumonia around site of malignant mass.

**CT Abd/Pelvis w/contrast -** I have reviewed the study dated 8/22/17 and it shows several suspicious lesions in the liver concerning for metastatic disease, enlarged superior mesenteric lymph nodes

**ECG** - I have interpreted and reviewed the study dated 8/22/17 and it shows: atrial fibrillation with RVR

**Pathology of right lung mass and liver lesions (outside record)** – FNA aspiration of right lung mass:

Tumor cells are immunopositive for TTF-1, cytokeratin AE1/AE3, and weakly positive for synaptophysin. CD4 is negative. These findings support the diagnosis of small cell carcinoma.

| **Assessment & Plan:** |
| --- |

Mr. Walt Gottfredson is a 67 y.o. male with a PMH significant for diabetes A1c 6.5+ on metformin, anxiety controlled on medications, atrial fibrillation not on anticoagulation (personal choice), GERD on PPI, tobacco abuse who presents for dyspnea, cough, hemoptysis for one month.

1. **Small Cell Lung Cancer, Extensive Stage, with metastasis to the Liver –**
2. **Systemic Inflammatory Response syndrome (SIRS) secondary to post obstructive pneumonia secondary to extensive small cell lung cancer -**
3. **Acute hypoxic respiratory failure secondary to SIRS -**
4. **Normocytic anemia -**
5. **Acute Kidney Injury secondary to SIRS -**
6. **Hyponatremia secondary to possible SIADH and malignancy -**
7. **Hypocalcemia without EKG changes -**
8. **Abnormal Liver Function Tests likely secondary to liver lesions -**
9. **Atrial Fibrillation with rapid ventricular response -**
10. **Severe protein-calorie malnutrition secondary to malignancy -**
11. **Uncontrolled Diabetes Mellitus without complications, A1C 7.6 -**
12. **GERD – controlled on PPI -**
13. **Tobacco abuse – education provided, pharmacologic intervention with 21mg nicotine patch**
14. **DVT Prophylaxis with enoxaparin**
