# Supplementary material for: An Interactive Multimodality Curriculum Teaching Medicine Residents About Oncologic Documentation and Billing
Source: MedEdPORTAL. 2018 Aug 30;14:10746. doi: 10.15766/mep_2374-8265.10746 (PMC6346345; doi:10.15766/mep_2374-8265.10746)
Supplement: Supplementary file 1 — A. Preintervention Survey.docx B. Blank H&P 1.docx C. Billing and Coding Lecture.pptx D. Blank H&P 2.docx E. Standardized Rubric.docx F. Postintervention Survey.docx G. H&P 1.docx H. H&P 2.docx I. Summary of Current Studies.docx [file mep-14-10746-s001.zip › H._H&P_2.docx]

**NAME:_____________________**

**Year (circle one)**

**PGY1 PGY2 PGY3**

| Template added by **Bosse, Raphael Charles, MD** at **09/18/17 1404**  Added by **Bosse, Raphael Charles, MD** at **09/18/17 1404**  Template added by **Bosse, Raphael Charles, MD** at **09/18/17 1404**  Template added by **Bosse, Raphael Charles, MD** at **09/18/17 1404**  Added by **Bosse, Raphael Charles, MD** at **09/18/17 1404**  Template added by **Bosse, Raphael Charles, MD** at **09/18/17 1404**  Template added by **Bosse, Raphael Charles, MD** at **09/18/17 1404**  Template added by **Bosse, Raphael Charles, MD** at **09/18/17 1404**  Template added by **Bosse, Raphael Charles, MD** at **09/18/17 1404**  Template added by **Bosse, Raphael Charles, MD** at **09/18/17 1404**  Added by **Bosse, Raphael Charles, MD** at **09/18/17 1404**  Template added by **Bosse, Raphael Charles, MD** at **09/18/17 1404**  Template added by **Bosse, Raphael Charles, MD** at **09/18/17 1404**  Template added by **Bosse, Raphael Charles, MD** at **09/18/17 1404**  Template added by **Bosse, Raphael Charles, MD** at **09/18/17 1404**  Added by **Bosse, Raphael Charles, MD** at **09/18/17 1404**  Template added by **Bosse, Raphael Charles, MD** at **09/18/17 1404**  Template added by **Bosse, Raphael Charles, MD** at **09/18/17 1404**  Template added by **Bosse, Raphael Charles, MD** at **09/18/17 1404**  Template added by **Bosse, Raphael Charles, MD** at **09/18/17 1404**  Template added by **Bosse, Raphael Charles, MD** at **09/18/17 1404**  Template added by **Bosse, Raphael Charles, MD** at **09/18/17 1404**  Template added by **Bosse, Raphael Charles, MD** at **09/18/17 1404**  Template added by **Bosse, Raphael Charles, MD** at **09/18/17 1404**  Template added by **Bosse, Raphael Charles, MD** at **09/18/17 1404**  Template added by **Bosse, Raphael Charles, MD** at **09/18/17 1404**  Template added by **Bosse, Raphael Charles, MD** at **09/18/17 1404**  Template added by **Bosse, Raphael Charles, MD** at **09/18/17 1404**  Template added by **Bosse, Raphael Charles, MD** at **09/18/17 1404**  Template added by **Bosse, Raphael Charles, MD** at **09/18/17 1404**  Added by **Bosse, Raphael Charles, MD** at **09/18/17 1404**  Added by **Bosse, Raphael Charles, MD** at **09/18/17 1404**  Added by **Bosse, Raphael Charles, MD** at **09/18/17 1404**  Added by **Bosse, Raphael Charles, MD** at **09/18/17 1404**  Added by **Bosse, Raphael Charles, MD** at **09/18/17 1404**  Added by **Bosse, Raphael Charles, MD** at **09/18/17 1404**  Added by **Bosse, Raphael Charles, MD** at **09/18/17 1404**  Added by **Bosse, Raphael Charles, MD** at **09/18/17 1404**  Added by **Bosse, Raphael Charles, MD** at **09/18/17 1404**  Added by **Bosse, Raphael Charles, MD** at **09/18/17 1404**  Template added by **Bosse, Raphael Charles, MD** at **09/18/17 1404**  Template added by **Bosse, Raphael Charles, MD** at **09/18/17 1404**  Template added by **Bosse, Raphael Charles, MD** at **09/18/17 1404**  Template added by **Bosse, Raphael Charles, MD** at **09/18/17 1404**  Template added by **Bosse, Raphael Charles, MD** at **09/18/17 1404**  Template added by **Bosse, Raphael Charles, MD** at **09/18/17 1404**  Template added by **Bosse, Raphael Charles, MD** at **09/18/17 1404**  Template added by **Bosse, Raphael Charles, MD** at **09/18/17 1404**  Added by **Bosse, Raphael Charles, MD** at **09/18/17 1404**  Template added by **Bosse, Raphael Charles, MD** at **09/18/17 1404**  Template added by **Bosse, Raphael Charles, MD** at **09/18/17 1404**  Template added by **Bosse, Raphael Charles, MD** at **09/18/17 1404**  Template added by **Bosse, Raphael Charles, MD** at **09/18/17 1404**  Template added by **Bosse, Raphael Charles, MD** at **09/18/17 1404**  Added by **Dang, Nam H, MD** at **09/19/17 0821**  **Department of Internal Medicine**  **History and Physical**  **Heme/Onc Team**    Admission Date and Time: 9/18/2017  8:25 AM  Primary Care Physician: John Doe, MD, PhD    **CC:** Scheduled chemotherapy, ICE cycle 2     \| **History of Present Illness:** \| \| --- \|     Ms. Irma Hurricane is a 21 y.o. female with a PMH significant for asthma, seasonal allergies, right atrial thrombus (anticoagulation with rivaroxaban) and stage IV A subcutaneous panniculitis like T cell lymphoma who presents for scheduled chemotherapy, ICE cycle 2.  The patient tolerated her previous cycle well, except for nausea and vomiting which was well controlled with anti-emetic agents. She has maintained good appetite and maintained her average weight as well. The patient recently had a right atrial thrombus diagnosed during previous hospitalization. She denies leg swelling, fevers/chills, night sweats, n/v, diarrhea, constipation, abdominal pain, chest pain/tightness. She had worsening urinary symptoms of dysuria prior to this admission and was found to have a UTI susceptible to macrobid.  She was first diagnosed in 2/3017 via biopsy which revealed subcutaneous panniculitis-like T-cell lymphoma. Bone marrow biopsy at that time revealed a hypocellular marrow (50%) and mild erythroid hyperplasia without overt evidence of lymphoma, abnormal T-cells population, or myeloblasts. She has undergone 3-cycles of EPOCH from June-July 2017, followed by cycle 1 of ICE salvage chemotherapy in August 2017. Following cycle 2, a CT will be done to evaluate for response, and following cycle 3 a PET will be completed, followed by a possible autologous bone marrow transplant.     \| **Review of Systems:** \| \| --- \|     Review of Systems  Constitutional: Negative for chills, diaphoresis, fever, malaise/fatigue and weight loss.  HENT: Negative for congestion, ear discharge, ear pain, hearing loss, nosebleeds, sinus pain, sore throat and tinnitus.  Eyes: Negative for blurred vision, double vision, photophobia, pain, discharge and redness.  Respiratory: Negative for cough, hemoptysis, sputum production, shortness of breath, wheezing and stridor.  Cardiovascular: Positive for leg swelling. Negative for chest pain, palpitations, orthopnea, claudication and PND.  Gastrointestinal: Positive for constipation. Negative for abdominal pain, blood in stool, diarrhea, heartburn, melena, nausea and vomiting.  Genitourinary: Negative for dysuria, flank pain, frequency, hematuria and urgency.  Musculoskeletal: Positive for joint pain. Negative for back pain, falls, myalgias and neck pain.  Skin: Negative for itching and rash.  Neurological: Positive for headaches. Negative for dizziness, tingling, tremors, sensory change, speech change, focal weakness, seizures, loss of consciousness and weakness.  Endo/Heme/Allergies: Negative for environmental allergies and polydipsia. Does not bruise/bleed easily.  Psychiatric/Behavioral: Negative for depression, hallucinations, memory loss, substance abuse and suicidal ideas. The patient is not nervous/anxious and does not have insomnia.     \| **Past Medical and Surgical History:** \| \| --- \|      \|  \| Past Medical History \| \| --- \| --- \| \| \|  \|  \|  \| \| --- \| --- \| --- \| \| **Past Medical History:** \| \| \| \| Diagnosis \| \| Date \| \|  \| Cancer \|  \| \|  \| *Lymphoma* \| \|   Asthma  Seasonal Allergies \| \|    Right atrial thrombus   \|  \| Past Surgical History \| \| --- \| --- \| \| \|  \|  \|  \|  \| \| --- \| --- \| --- \| --- \| \| **Past Surgical History:** \| \| \| \| \| Procedure \| \| Laterality \| Date \| \| • \| WISDOM TOOTH EXTRACTION \|  \|  \| \| \|          \| **Social History:** \| \| --- \|      \| **Social History** \| \| --- \|      \|  \|  \|  \|  \| \| --- \| --- \| --- \| --- \| \| Social History \| \| \| \| \| • \| Marital status: \| \| Married \| \|  \|  \| Spouse name: \| N/A \| \| • \| Number of children: \| \| N/A \| \| • \| Years of education: \| \| N/A \|      \|  \|  \|  \| \| --- \| --- \| --- \| \| Occupational History \| \| \| \| • \| Administrative Assistant \|  \|      \|  \|  \|  \|  \| \| --- \| --- \| --- \| --- \| \| Social History Main Topics \| \| \| \| \| • \| Smoking status: \| \| Never Smoker \| \| • \| Smokeless tobacco: \| \| Never Used \| \| • \| Alcohol use \| \| No \| \| • \| Drug use: \| \| No \| \| • \| Sexual activity: \| \| Yes \| \|  \|  \| Partners: \| Male \|      \|  \|  \|  \| \| --- \| --- \| --- \| \| Other Topics \| \| Concern \| \| • \| None \| \|      \|  \|  \| \| --- \| --- \| \| Social History Narrative \| \| \|  \| *Lives in Pensacola, FL with husband.  Originally from Nevada. Works in a local office.* \|  \| **Family History:** \| \| --- \|      \|  \| Family History \| \| --- \| --- \| \| \|  \|  \|  \|  \|  \| \| --- \| --- \| --- \| --- \| --- \| \| **Family History** \| \| \| \| \| \| Problem \| \| \| Relation \| Age of Onset \| \| • \| Skin Cancer \| \| Other \|  \| \|  \|  \| *cousin with unknown type skin cancer* \| \| \| \| • \| Lymphoma \| \| Sister \| 20 \| \| • \| Rheumatoid Arthritis \| \| Mother \|  \| \| • \| Ovarian Cancer \| \| Maternal Grandmother \|  \| \| • \| Colon Cancer \| \| Paternal Grandfather \|  \| \| \|     I have reviewed the past medical, past surgical, family and social history.     \| **Allergies:** \| \| --- \|     No Known Allergies       \| **Home Medications:** \| \| --- \|      \|  \|  \|  \|  \| \| --- \| --- \| --- \| --- \| \| Medication \| Sig \| Start Date \| End Date \| \| **rivaroxaban (XARELTO) 15 MG Tablet** \| **Take 1 tablet by mouth 2 times daily (with meals).** \| **8/17/17** \|  \| \| **sulfamethoxazole-trimethoprim (BACTRIM DS,SEPTRA DS) 800-160 MG Tablet** \| **Take 1 tablet by mouth three times a week.** \| **6/19/17** \|  \| \| **norgestimate-ethinyl estradiol (SPRINTEC 28) 0.25-35 MG-MCG Tablet** \| **Take 1 tablet by mouth daily.** \| **2/23/17** \|  \| \| **ondansetron (ZOFRAN) 4 MG Tablet** \| **Take 1 tablet by mouth every 8 hours as needed. Take up to 3 times a day for nausea** \| **6/19/17** \|  \| \| **prochlorperazine (COMPAZINE) 10 MG Tablet** \| **Take 1 tablet by mouth every 6 hours as needed. Take days 4 to 6** \| **8/30/17** \|  \| \| **promethazine (PHENERGAN) 25 MG Tablet** \| **Take 0.5-1 tablets by mouth every 6 hours as needed for nausea or vomiting.** \| **6/19/17** \| **9/18/17** \| \| **senna-docusate (DOC-Q-LAX) 8.6-50 MG Tablet** \| **Take 2 tablets by mouth daily.** \| **7/10/17** \|  \|      \|  \| Current Medications \| \| --- \| --- \| \| \|  \|  \|  \|  \|  \|  \| \| --- \| --- \| --- \| --- \| --- \| --- \| \| Medication \| \| Dose \| Route \| Frequency \| Last Rate \| \| • \| 0.9 % NaCl infusion \|  \| Intravenous \| Continuous \|  \| \| • \| Active Chemo Patient 1 each \| 1 each \| Other \| PRN \|  \| \| • \| [START ON 9/19/2017] CARBOplatin (PARAPLATIN) 680 mg in D5W 250 mL chemo infusion \| 680 mg \| Intravenous \| Once \|  \| \| • \| dexamethasone (DECADRON) tablet 8 mg \| 8 mg \| Oral \| Q12H \|  \| \| • \| docusate sodium (COLACE) capsule 100 mg \| 100 mg \| Oral \| BID PRN \|  \| \| • \| etoposide (VEPESID) 190 mg in 0.9 % NaCl 500 mL chemo infusion \| 100 mg/m2 (Treatment Plan Recorded) \| Intravenous \| Q24H \|  \| \| • \| [START ON 9/19/2017] ifosfamide (IFEX) 9,650 mg, mesna (MESNEX) 9,650 mg in 0.9 % NaCl 1,000 mL chemo infusion \| 5,000 mg/m2 (Treatment Plan Recorded) \| Intravenous \| Once (infusion) \|  \| \| • \| LORazepam (ATIVAN) injection 1 mg \| 1 mg \| Intravenous \| Q6H PRN \|  \| \| • \| LORazepam (ATIVAN) tablet 1 mg \| 1 mg \| Oral \| Q6H PRN \|  \| \| • \| [START ON 9/20/2017] mesna (MESNEX) 4,830 mg in 0.9 % NaCl 500 mL IV \| 2,500 mg/m2 (Treatment Plan Recorded) \| Intravenous \| Once (infusion) \|  \| \| • \| norgestimate-ethinyl estradiol (ESTARYLLA) 0.25-0.35 mg-mcg per tablet 1 \| 1 tablet \| Oral \| daily \|  \| \| • \| OLANZapine (ZyPREXA Zydis) disintegrating tablet 5 mg \| 5 mg \| Oral \| Nightly \|  \| \| • \| ondansetron (ZOFRAN) tablet 4 mg \| 4 mg \| Oral \| Q8H PRN \|  \| \| • \| ondansetron (ZOFRAN-ODT) disintegrating tablet 8 mg \| 8 mg \| Oral \| Q12H \|  \| \| • \| polyethylene glycol (MIRALAX) packet 17 g \| 17 g \| Oral \| Q12H PRN \|  \| \| • \| promethazine (PHENERGAN) 25 mg in 0.9 % NaCl 25 mL IVPB \| 25 mg \| Intravenous \| Q6H PRN \|  \| \| • \| promethazine (PHENERGAN) tablet 25 mg \| 25 mg \| Oral \| Q6H PRN \|  \| \| • \| rivaroxaban (XARELTO) \| 15 mg \| Oral \| BID W/ Meals \|  \| \| \|          \| **Objective:** \| \| --- \|      \| **Vital Signs: Last Filed** \| **Vitals Signs: 24 Hour Range** \| \| --- \| --- \| \| Temp: 37 °C (98.6 °F) (09/18 1319) \| Temp:  [36.9 °C (98.4 °F)-37 °C (98.6 °F)] \| \| Pulse: 85 (09/18 1319) \| Pulse:  [85-87] \| \| BP: 129/57 (09/18 1319) \| BP: (114-129)/(57-62) \| \| Resp: 16 (09/18 1319) \| Resp:  [15-16] \| \| SpO2: 100 % (09/18 1319) \| SpO2:  [99 %-100 %] \|     **Weight** **:**   \|  \|  \| \| --- \| --- \| \| **Wt Readings from Last 3 Encounters:** \| \| \| 09/18/17 \| 81.8 kg (180 lb 5.4 oz) \| \| 09/14/17 \| 81.6 kg (179 lb 12.8 oz) \| \| 09/01/17 \| 79.4 kg (175 lb 0.7 oz) \|    Body mass index is 30.14 kg/(m^2).    **Constitutional:** well developed, no distress  **Eyes:** extra-ocular movements in-tact, no scleral icterus, PERRL  **Ears/Nose/Throat/Mouth/Neck:** moist mucous membranes without lesions, neck soft and supple  **Cardiovascular:** normal rate, regular rhythm, no murmur, rub or gallop, 2+ radial pulses, no pitting edema. **Swelling present in right calf > left, no erythema or tenderness present.**  **Respiratory:** clear to auscultation with good air movement bilaterally  **Gastrointestinal:** +BS, abdomen soft, non-tender and non-distended  **Musculoskeletal:** no joint swelling or clubbing  **Skin:** normal turgor, no jaundice. **Right PORT catheter in place, no erythema or swelling noted.**  **Neurological:** moves all extremities, no facial asymmetry, CNII-XII grossly intact, no focal deficits  **Psychiatric:** A&Ox3, normal mood and affect    **Access:**   \|  \|  \|  \| \| --- \| --- \| --- \| \| Port A Cath Single Chamber  08/28/17 Power Right Chest (Active) \| \| \| \| Site Assessment \| Clean;Dry;Intact \| 9/18/2017  8:40 AM \| \| Line Status \| Blood return noted;Capped;Flushed;Heparin lock;Valve changed \| 9/18/2017  8:40 AM \| \| Line Care \| Connections checked and tightened \| 9/18/2017  8:40 AM \| \| Dressing Type \| Transparent;Antimicrobial disc \| 9/18/2017  8:40 AM \| \| Dressing Status \| Changed/New \| 9/18/2017  8:40 AM \| \| Dressing Change Due \| 09/25/17 \| 9/18/2017  8:40 AM \| \| Flush Performed \| Yes \| 9/18/2017  8:40 AM \| \| Date to be Reflushed \| 09/18/17 \| 9/18/2017  8:40 AM \| \| Needle Size \| 20g 3/4" \| 9/18/2017  8:40 AM \| \| Needle insert date \| 09/18/17 \| 9/18/2017  8:40 AM \| \| Needle change date \| 09/25/17 \| 9/18/2017  8:40 AM \| \| Valve Change Due (GNV ONLY) \| 09/20/17 \| 9/18/2017  8:40 AM \|     PIV: Yes  Foley: No     \| **Data Review:** \| \| --- \|     I have reviewed the following laboratory studies:      **Recent Labs**   \| **Lab** \| **09/14/17 0934** \| \| --- \| --- \| \| NA \| 138 (136-145 mmol/L) \| \| K \| 4.5 (3.3-5.1 mmol/L) \| \| CL \| 104 (98-107 mmol/L) \| \| CO2 \| 30 (22-30 mmol/L) \| \| BUN \| 10 (6-21 mg/dL) \| \| CREATININE \| 0.82 (0.38-1.02 mg/dL) \| \| GLU \| 82 (65-99 mg/dL) \| \| CALCIUM \| 9.6 (8.4-10.2mg/dL) \| \| MG \| 2.1 (1.5-2.8 mg/dL) \| \| PHOS \| 4.0 (2.7-4.5 mg/dL) \|         **Recent Labs**   \| **Lab** \| **09/14/17 0934** \| \| --- \| --- \| \| TPROT \| 7.0 (6.4-8.3 g/dL) \| \| ALB \| 4.1 (3.5-5.2 g/dL) \| \| AST \| 19 (0-37 IU/L) \| \| ALT \| 17 (0-35 IU/L) \| \| TBILI \| 0.2 (0.0-1.0 mg/dL) \| \| ALKPHOS \| 60 (40-150 IU/L) \|         **Recent Labs**   \| **Lab** \| **09/14/17 0934** \| \| --- \| --- \| \| WBC \| 6.7 (3.0-10.0 thou/cu mm) \| \| HGB \| 12.0 (12.0-16.0 g/dL) \| \| HCT \| 36.1 (35.0-45.0%) \| \| PLATCOUNT \| 127* (150-450 thou/cu mm) \|      \|  \|  \|  \|  \| \| --- \| --- \| --- \| --- \| \| **Lab Results** \| \| \| \| \| Component \| \| Value \| Date/Time \| \|  \| TROPONINI \| <0.03 (<0.04 ng/mL) \| 08/16/2017 08:54 PM \| \|  \| TROPONINI \| <0.03 (<0.04 ng/mL) \| 08/16/2017 03:31 PM \| \|  \| TROPONINI \| <0.03 (<0.04 ng/mL) \| 06/18/2017 11:17 PM \|         **Micro:**   Blood cultures No Growth    **Imaging:**  **9/18/17 US DVT:**  Negative bilateral lower extremity deep venous US for DVT. No evidence of DVT of  either lower extremity.    None this admission.    8/1/17 CT Chest-Abdomen-Pelvis:  IMPRESSION:    1. Soft tissue lesion in the right atrium, suspicious for thrombus. Recommend further  evaluation with echocardiography.    2. Unchanged inguinal lymphadenopathy, previously shown to be non FDG avid.    3. Slight asymmetry of the left infraspinatus musculature in the area of prior abnormal FDG  activity without discrete mass.    4. Subcentimeter right lower lobe pulmonary nodules which can be followed on subsequent exams.   \| **Assessment & Plan:** \| \| --- \|     Ms. Irma Hurricane is a 21 y.o. female with a PMH significant for asthma, seasonal allergies, right atrial thrombus (anticoagulation with rivaroxaban) and stage IV A subcutaneous panniculitis like T cell lymphoma who presents for scheduled chemotherapy, ICE cycle 2.   1. T-cell lymphoma, subcutaneous panniculitis – cont ICE 2. Right atrial thrombus on anticoagulation with rivaroxaban 3. Lower extremity edema – Doppler negative 4. Cystits - abx 5. Asthma/COPD – not on home O2 6. Seasonal allergies - stable 7. Constipation – will prescribe bowel medications 8. Joint Pain – will eval for RA/Gout 9. Headache – no ominous features 10. Thrombocytopenia – due to chemotx and disease 11. Pulmonary Nodules – follow on CT scan |
| --- | --- | --- | --- | --- | --- | --- | --- | --- | --- | --- | --- | --- | --- | --- | --- | --- | --- | --- | --- | --- | --- | --- | --- | --- | --- | --- | --- | --- | --- | --- | --- | --- | --- | --- | --- | --- | --- | --- | --- | --- | --- | --- | --- | --- | --- | --- | --- | --- | --- | --- | --- | --- | --- | --- | --- | --- | --- | --- | --- | --- | --- | --- | --- | --- | --- | --- | --- | --- | --- | --- | --- | --- | --- | --- | --- | --- | --- | --- | --- | --- | --- | --- | --- | --- | --- | --- | --- | --- | --- | --- | --- | --- | --- | --- | --- | --- | --- | --- | --- | --- | --- | --- | --- | --- | --- | --- | --- | --- | --- | --- | --- | --- | --- | --- | --- | --- | --- | --- | --- | --- | --- | --- | --- | --- | --- | --- | --- | --- | --- | --- | --- | --- | --- | --- | --- | --- | --- | --- | --- | --- | --- | --- | --- | --- | --- | --- | --- | --- | --- | --- | --- | --- | --- | --- | --- | --- | --- | --- | --- | --- | --- | --- | --- | --- | --- | --- | --- | --- | --- | --- | --- | --- | --- | --- | --- | --- | --- | --- | --- | --- | --- | --- | --- | --- | --- | --- | --- | --- | --- | --- | --- | --- | --- | --- | --- | --- | --- | --- | --- | --- | --- | --- | --- | --- | --- | --- | --- | --- | --- | --- | --- | --- | --- | --- | --- | --- | --- | --- | --- | --- | --- | --- | --- | --- | --- | --- | --- | --- | --- | --- | --- | --- | --- | --- | --- | --- | --- | --- | --- | --- | --- | --- | --- | --- | --- | --- | --- | --- | --- | --- | --- | --- | --- | --- | --- | --- | --- | --- | --- | --- | --- | --- | --- | --- | --- | --- | --- | --- | --- | --- | --- | --- | --- | --- | --- | --- | --- | --- | --- | --- | --- | --- | --- | --- | --- | --- | --- | --- | --- | --- | --- | --- | --- | --- | --- | --- | --- | --- | --- | --- | --- | --- | --- | --- | --- | --- | --- | --- | --- | --- | --- | --- | --- | --- | --- | --- | --- | --- | --- | --- | --- | --- | --- | --- | --- | --- | --- | --- | --- | --- | --- | --- | --- | --- | --- | --- | --- | --- | --- | --- | --- | --- | --- | --- | --- | --- | --- | --- | --- | --- | --- | --- | --- | --- | --- | --- | --- | --- | --- | --- | --- | --- | --- | --- | --- | --- | --- | --- | --- | --- | --- | --- | --- | --- | --- | --- | --- | --- | --- | --- | --- | --- | --- | --- | --- | --- | --- | --- | --- | --- | --- | --- | --- | --- | --- | --- | --- | --- | --- | --- | --- | --- | --- | --- | --- | --- | --- | --- | --- | --- | --- | --- | --- | --- | --- | --- | --- | --- | --- | --- | --- | --- | --- | --- | --- | --- | --- | --- | --- | --- | --- | --- | --- | --- | --- | --- | --- | --- | --- | --- | --- | --- | --- | --- | --- | --- | --- | --- | --- | --- | --- | --- | --- | --- | --- | --- | --- | --- | --- | --- | --- | --- | --- | --- | --- | --- | --- | --- | --- | --- | --- | --- | --- |
