# Supplementary material for: An Interactive Multimodality Curriculum Teaching Medicine Residents About Oncologic Documentation and Billing
Source: MedEdPORTAL. 2018 Aug 30;14:10746. doi: 10.15766/mep_2374-8265.10746 (PMC6346345; doi:10.15766/mep_2374-8265.10746)
Supplement: Supplementary file 1 — A. Preintervention Survey.docx B. Blank H&P 1.docx C. Billing and Coding Lecture.pptx D. Blank H&P 2.docx E. Standardized Rubric.docx F. Postintervention Survey.docx G. H&P 1.docx H. H&P 2.docx I. Summary of Current Studies.docx [file mep-14-10746-s001.zip › I._Summary_of_Current_Studies.docx]

Appendix I – Table summarizing current publications addressing medical education regarding documentation.

| Reference | Total Participants | Learners (Department) | Setting | Intervention | Result |
| --- | --- | --- | --- | --- | --- |
| Ghaderi et. al^1^ | 12 | Residents (Plastic Surgery) | Outpatient | 3 didactic sessions (20 minutes each), visual aids in clinic (for review of evaluation and management) | Significant increase in billing, and increase in evaluation and management codes |
| Liang e.t al^3^ | 11 | Residents (Neurology) | Outpatient | 1 hour lecture, email “paystubs” | Under-documentation was significantly reduced |
| Ferzandipour et. al^4^ | 19 | Residents (Internal Medicine, Obstetrics and Gynecology, Surgery) | Inpatient | Guidelines for “recording diagnostic information” taught in a 5-hour lecture | No improvement in quality and accuracy of recording |
| Momin et. al^8^ | 29 | 12 Residents  17 Attendings (Otolaryngology) | Inpatient | “education on billing and coding” | Case mix index increased after intervention but not significantly. Percentage of patients with documented complication, comorbidity, severity of illness 3 or 4, or mortality score of 3 or 4 increased significantly |
| Kim et. al^9^ | Unspecified | Resident physicians (unspecified) | Unspecified | “Real time feedback” for residents | Proportion of target diagnoses, median severity of illness, and complication code capture increased significantly; no statistically significant increase in risk of mortality |
| Varacallo et. al^10^ | 32 | Residents (Orthopedic Surgery) | Unspecified | 45 minute lecture followed by posttest examination, exam given by faculty blinded to the lecture, residents then asked to self-rate comfort with documentation | Teaching session improved test scores significantly |
| Rosenbaum et. al^13^ | Unspecified | Clinicians included Attendings, Fellows, Residents, Physician Assistants, and Nurse Practitioners (Neurosurgery) | Inpatient | “Educational lectures” given by “physician champions” | Case mix index, normalized case mix index, MS-DRG, expected length of stay were improved after intervention |
| Spellberg et. al^14^ | Unspecified | Residents (Internal Medicine) | Inpatient | Lectures, and a pocket card | Median quarterly complication codes and major complication code capture rate increased significantly. Median expected mortality and median quarterly case mix index increased significantly |
| Sprtel et. al^15^ | Unspecified | Residents (Unspecified) | Unspecified | Documentation lecture, pre-trial and post-trial surveys | Billable income increased significantly |
| Hirch et. al^17^ | Unspecified | Residents (Emergency Medicine) | Outpatient/ER | Document on billing and coding, lecture on coding “given by faculty”, “one-on-one session with the faculty member” for chart audits | Residents reporting high level of comfort with billing and coding |
| Lee et. al^19^ | 20 | Residents (Internal Medicine) | Unspecified | Module introducing learners to ICD-10 coding (given as three 50-minute sessions), pre-test, post-test, simulated patient encounters | 88% learners reported this exercise helpful, and significant improvement in posttest scores |
| Miller et. al^20^ | Unspecified | Residents (Family Medicine) | Outpatient | Color-coded audit form, PowerPoint presentation, and 12 weekly maintenance-of-skills scenarios, groups asked to discuss level of service designation and reach a consensus. Participants then asked to complete posttest and evaluation | No significant difference was observed in ability to choose correct level of service |
